# Exploring the Anti-Inflammatory Potential of Saudi Propolis Through Phytochemical Characterization, Molecular Docking, and Dynamic Simulation

**DOI:** 10.3390/pharmaceutics18091050

**Published:** 2026-08-24

**Authors:** Hanan Aati, Jawaher H. Alqahtani, Areej Al-Taweel, Sultan Y. Aati

**Affiliations:** 1 Department of Pharmacognosy, College of Pharmacy, King Saud University, P.O. Box 22452, Riyadh 11495, Saudi Arabia; jalqahtani@ksu.edu.sa (J.H.A.); amaltaweel@ksu.edu.sa (A.A.-T.); 2 Dental Health Department, College of Applied Medical Sciences, King Saud University, Riyadh P.O. Box 10219, Saudi Arabia; sati@ksu.edu.sa

**Keywords:** propolis extracts, DPPH, ABTS, COX-1, COX-2, molecular docking, LC/MS, molecular dynamics

## Abstract

**Background/Objectives**: Propolis is a resinous natural product rich in phenolic acids and flavonoids, recognized in ethnopharmacology for its antioxidant and anti-inflammatory properties. This study aimed to identify the most bioactive Saudi propolis extract using a bioassay-guided strategy and investigate its chemical profile and mechanistic anti-inflammatory potential. **Methods**: Four propolis extracts (P1–P4) collected from different regions of Saudi Arabia were evaluated for their antioxidant (DPPH and ABTS) and anti-inflammatory (COX-1 and COX-2) activities. The most active extract was profiled using liquid chromatography–mass spectrometry (LC–MS), followed by molecular docking and molecular dynamics simulations. **Results**: Among all samples, P3 demonstrated the strongest antioxidant activity, with IC_50_ values of 25.84 ± 0.96 µg/mL (DPPH) and 32.30 ± 1.20 µg/mL (ABTS), comparable to ascorbic acid (27.45 ± 1.42 and 21.22 ± 0.79, respectively). P3 also exhibited potent and selective COX-2 inhibition with an IC_50_ of 6.19 ± 0.21 µg/mL (relative to celecoxib, IC_50_ 0.681 ± 0.02 as positive control). LC–MS analysis identified 26 secondary metabolites. Computational studies ranked kaempferol as forming the most conformationally stable COX-2 complex among the ligands examined, including the reference inhibitor, on the basis of molecular dynamics descriptors of pose persistence and conformational confinement. **Conclusions**: Saudi propolis P3 is a potent source of bioactive compounds with strong antioxidant and selective COX-2 inhibitory activity. These findings highlight its anti-inflammatory potential and suggest its value as a natural lead for developing safer therapeutics targeting inflammation-related chronic diseases.

## 1. Introduction

Propolis, commonly known as “bee glue” [1,2], is a natural resinous substance formed by honeybees’ salivary secretion (*Apis mellifera* L.) in combination with plant exudates, mainly leaf buds with beeswax ranging from yellowish-green to dark brown in color with a characteristic aromatic odor [2]. The word “propolis” originates from the Greek words “pro”, meaning “in defense of”, and “polis”, meaning “city” [3], highlighting its essential role as a protective barrier against pathogens and external threats [4,5]. Propolis plays a crucial role in the nest structural maintenance by sealing cracks and insulating the hives, ensuring the health and stability of bee colonies [6,7]. The diversity of compositions and bioactivity is mainly related to regional flora from which the plant resins are collected [8,9]. Propolis has been utilized since at least 300 BC, employed by Egyptians for mummification and as a topical cream for wounds and ulcers. In Russia, it was known as “Russian penicillin”. The National Food Institute (INAL) of Argentina officially classified propolis as a dietary supplement in 1995 [10,11]. Crude propolis is nearly composed of 50% resin and balsam, 30% beeswax, 10% volatile oils, 5% pollen, and 5% organic and inorganic compounds with predominant compounds like flavonoids, phenols, sugars, amino acids, vitamins and minerals [9].

Propolis is famous in traditional medicine for its wide range of therapeutic effects against inflammation, microbial infections, hepatotoxicity, cardiovascular diseases and various forms of cancer [4,12]. Extensive research has demonstrated the anticancer potential of propolis and its phenolic constituents through several mechanisms [5]. Furthermore, its antioxidant activity has been confirmed by various chemical assays, including DPPH radical-scavenging and superoxide anion-scavenging tests [5,13].

Flavonoids and phenolic compounds are among the most extensively studied constituents of propolis, recognized for their strong antioxidant properties and their ability to inhibit lipid oxidation through radical-scavenging mechanisms. Moreover, the presence of flavonoids and other phenolic substances has been associated with a protective role against the development of cancer and cardiovascular diseases [8]. Likewise, Suran et al. (2021) noted that propolis extracts and their bioactive components can stimulate the nuclear factor erythroid 2-related factor 2–antioxidant response element (Nrf2–ARE) intracellular antioxidant signaling pathway, boosting the expression of antioxidant genes [14].

Inflammation is a complex process linked to various conditions, including arthritis, asthma, and psoriasis. Cyclooxygenase (COX), the key enzyme in the eicosanoid biosynthetic pathway, has two isoforms: cyclooxygenase 1 (COX-1) and cyclooxygenase 2 (COX-2) [15]. COX-1 is responsible for generating cytoprotective prostaglandins in the gastrointestinal tract and pro-aggregatory thromboxanes in blood platelets. COX-2 is an enzyme that can be induced in reaction to various pro-inflammatory substances like TNFα, IL-6, IL-1, carrageenan, and histamine. The anti-inflammatory effects of non-steroidal anti-inflammatory drugs (NSAIDs), such as aspirin and indomethacin, are limited as aspirin preferentially inhibits COX-1 over COX-2, resulting in an ulcerogenic adverse effect [16]. One well-known anti-inflammatory NSAID is celecoxib. Nevertheless, celecoxib and other coxibs can lead to severe heart or circulatory issues like heart attack or stroke, particularly with prolonged use [16,17]. Consequently, the creation of new compounds exhibiting anti-inflammatory effects with a better safety profile remains critically significant.

All these findings regarding the biological properties of propolis motivated us to investigate the chemical ingredients and the biological activities (antioxidant and anti-inflammatory activities) of four different propolis extracts collected from various regions across Saudi Arabia (Riyadh [P1], Al-Madinah Al-Manawarah [P2], Fayfa Mountain [P3], and Al-Baha Mountain [P4]).

This study adopted a bioassay-guided strategy to evaluate the therapeutic potential of four Saudi Arabian propolis extracts. First, the extracts were comprehensively screened for antioxidant capacity (using DPPH and ABTS assays) and anti-inflammatory activity (using COX-1 and COX-2 inhibition assays). The most active extract was then chemically profiled via LC-MS/MS to identify its primary active constituents. To investigate the molecular mechanisms driving these biological results, computer-aided drug design (CADD) approaches—including molecular docking and molecular dynamics simulations—were employed. Utilizing these computational methods allows for the strategic prioritizing and filtering of natural compounds, providing a clear structural rationale for their potential as innovative drug candidates while significantly reducing downstream experimental resources. Molecular docking was employed to assess the binding process and strength of a small molecule (ligand) with a target macromolecule to predict the main active constituents that may be responsible for the anti-inflammatory activities of the most active propolis extract [18].

To achieve the target of our study, the following was performed: (A) The antioxidant activities of the four extracts under study were measured using two methods: DPPH and ABTS. (B) The anti-inflammatory activities were investigated using COX inhibition assays. (C) Metabolite profiling of the most active extract was evaluated using LC/MS technique. (D) Molecular docking and dynamics simulations, in addition to trajectory analysis, were achieved to predict the active constituents that may be responsible for the biological activities.

## 2. Materials and Methods

### 2.1. Sample Collection

Propolis samples were collected from four regions of Saudi Arabia (Riyadh [P1], Al-Madinah Al-Munawarah [P2], Fayfa Mountain; 17.255° N, 43.102° E [P3], and Al-Baha Mountain; 19.9486000° N, 41.4855000° E [P4]) in January 2025 from *Apis mellifera jemenitica* colonies. Samples were manually harvested using sterile tools, stored at low temperature, and winter-collected to ensure stability and minimize environmental variation. Differences among samples were attributed to geographical origin and surrounding resinous flora. Apiaries were surrounded by diverse plant sources, including Moringa, Tecoma, Ziziphus, *Acacia gerrardii*, Conocarpus, *Anastatica hierochuntica* L., *Phoenix dactylifera* L., *Tamarix aphylla* (L.) H. Karst., and *Eucalyptus* spp.

### 2.2. Propolis Extraction

Propolis extracts were first grounded and then extracted with 70% ethanol at a ratio of 30 g of propolis per 100 mL of solvent. The extraction was carried out at room temperature, protected from light, and under moderate agitation for one week, following the method described by Sforcin, et al. (2005) [19]. Afterward, the extracts were filtered, and the dry weight was determined by completely evaporating the solvent from a 1 mL aliquot, yielding a concentration of 140 mg of propolis per mL. Specific dilutions were subsequently prepared for each assay (DPPH assay, ABTS assay, COX-1 and COX-2 assays).

### 2.3. Antioxidant Assays

#### 2.3.1. DPPH Assay

The antioxidant activity of the propolis samples was evaluated using the 1,1-diphenyl-2-picrylhydrazyl (DPPH) radical-scavenging assay, following the method described by Islam et al. (2024) [18]. A DPPH solution was prepared by dissolving 0.004 g of DPPH reagent in 100 mL of methanol. This solution was subsequently mixed with various concentrations of the propolis extracts (300, 150, 100, 50, 25, 12.5, and 6.25 µg/mL). After incubation at 25 °C for 15 min, the absorbance of each sample was recorded at 517 nm.

Ascorbic acid was used as a standard antioxidant (positive control), and its calibration curve (300-6.25 µg/mL) was employed to calculate the IC_50_ values of the samples the concentration required to scavenge 50% of DPPH radicals. The DPPH radical-scavenging activity (%) was calculated using the following equation:

**DPPH Scavenging Activity (%) =** (A_control_ − As_ample_)/(A_control_ × 100)

where A_control_ is the absorbance of the DPPH solution without propolis extracts, and A_sample_ is the absorbance in the presence of the propolis extracts.

The antioxidant capacity of the samples was further expressed as ascorbic acid-Equivalent Antioxidant Capacity (TEAC), calculated using the following formula:

**TEAC =** IC_50_ (ascorbic acid)/IC_50_ (Sample)

All measurements were performed in triplicate to ensure reproducibility and statistical reliability [20,21].

#### 2.3.2. ABTS Antioxidant Assay

The antioxidant activity of the samples was evaluated using the ABTS radical cation decolorization assay, following the method described in [22]. To generate the ABTS^•+^ working solution, 5 mL of 7 mM ABTS stock solution was mixed with 88 µL of 140 mM potassium persulfate and incubated in the dark at room temperature for 12 to 16 h. The resulting solution was then diluted with distilled water in a 1:3 (*v*/*v*) ratio to obtain an absorbance of 0.7 at 734 nm.

Propolis extracts (P1, P2, P3 and P4) were mixed with the ABTS reagent in a 1:1 ratio and incubated at 37 °C in the dark for 6 min. The absorbance of each mixture was measured at 734 nm using a UV–visible spectrophotometer. All measurements were carried out in triplicate. Ascorbic acid was used as the standard reference antioxidant.

The IC_50_ values, which represent the attenuation percentage values, were calculated using the following formula:

**ABTS Scavenging Activity (%) =** (A_control_ − A_sample_)/(A_control_) × 100

where A_control_ is the absorbance of the ABTS solution without propolis extracts, and A_sample_ is the absorbance in the presence of the propolis extracts.

### 2.4. COX Inhibition Assay

Enzymatic in vitro assays using COX-1 or COX-2 (both Sigma-Aldrich, St. Louis, MO, USA) were performed to test the inhibitory activities of the tested propolis extracts (P1, P2, P3 and P4). In vitro COX-1 and COX-2 inhibition was evaluated using the same fluorometric screening assay [23]. Six distinct concentrations, e.g., 150, 100, 50, 25, 12.5, and 6.25 μg/mL, for each propolis extract (P1–P4) and the reference control (Celecoxib, Sigma-Aldrich, St. Louis, MO, USA), were performed. Test inhibitors were dissolved in an appropriate solvent such as dimethyl sulfoxide (DMSO) and subsequently diluted to 10 times the desired test concentration using COX Assay Buffer. Then, 10 μL of the diluted inhibitor or assay buffer was added into designated wells to serve as either the sample screen (S) or the enzyme control (EC) without inhibitor. Inhibitor control (IC) was prepared by mixing 2 μL of SC560 with 8 μL of COX Assay Buffer and adding the mixture into a separate well.

Since the solvents used to dissolve the inhibitors may interfere with enzyme activity, it is important to include a solvent control well containing the same final concentration of the solvent as in the test samples to distinguish between the effects of the solvent and those of the inhibitor itself.

The COX Cofactor was freshly diluted at 1:200 by mixing 2 μL of the stock with 398 μL of COX Assay Buffer. The Arachidonic Acid solution was prepared by combining 5 μL of Arachidonic Acid with 5 μL of NaOH followed by vortexing and then further diluted 1:10 with 90 μL of distilled water. Eighty microliters of the prepared reaction mix was added to each well. A multi-channel pipette was used to dispense 10 μL of the diluted Arachidonic Acid/NaOH solution to initiate the enzymatic reaction across all wells to initiate all the reactions at the same time.

The diluted COX Cofactor was stable for up to one hour at room temperature and the diluted Arachidonic Acid/NaOH solution was stable for at least one hour when kept on ice. Both solutions were prepared freshly before use and not stored. The microplate reader was preset prior to starting the assay to avoid delay in measurement after reaction initiation.

Fluorescence was measured kinetically at 25 °C over a period of 5 to 10 min using excitation and emission wavelengths of 535 and 587 nm, respectively. Two time points (T1 and T2) were selected within the linear range of the fluorescence signal and corresponding readings (RFU1 and RFU2) were obtained. The slope for each sample including the enzyme control was calculated using the formula: Slope = (RFU2 − RFU1)/(T2 − T1). Relative inhibition was determined using the following equation:

**% Relative Inhibition =** [(Slope of EC − Slope of Sample)/Slope of EC] × 100

### 2.5. Statistical Analysis

Data are displayed as the mean ± standard deviation (SD). Mean comparisons were executed using one-way analysis of variance (ANOVA), followed by Tukey’s post hoc multiple-comparison test. A difference with a *p*-value lower than 0.05 was considered statistically significant. The IC_50_ values were calculated from the calibration curve determined by linear regression.

### 2.6. LC-MS Analysis

LC-MS/MS data processing and annotation metrics: Raw chromatograms were evaluated programmatically using an automated Molecular Feature Extraction (MFE) algorithm. Chromatographic feature tracking was restricted to true compound peaks exhibiting clean widths between 0.028 and 1.897 min, a strict signal height ceiling of 25,000 counts, and an explicit isotope cluster constraint cross-referenced directly against parental base peaks.

Chemical structural annotations were authenticated by matching high-resolution experimental mass-to-charge ratios (*m*/*z*) across alternating ESI source polarities against standard phytochemical databases. Structural identity validation was restricted to components displaying an MFE confidence quality score between 70% and 100%. Relative concentrations were derived as percent volume fractions (% vol.) using integrated peak areas verified across multiple replicates to establish high quantitative reproducibility.

### 2.7. Protein and Ligand Preparation

The crystallographic structures of COX-1 in complex with celecoxib (PDB ID: 3KK6, resolution 2.75 Å) [24] and of murine COX-2 in complex with rofecoxib (PDB ID: 5KIR, resolution 2.34 Å) [25] were retrieved from the RCSB Protein Data Bank and used as the molecular targets for both the docking and the MD simulation phases of this work. Each structure was prepared by removing crystallographic water molecules, ions, and co-purified buffer components other than the heme cofactor, which was retained in its native position to preserve the integrity of the cyclooxygenase channel. Hydrogen atoms were added at pH 7.4, and the protonation state of histidine residues, including the heme-coordinating proximal histidine, was assigned by inspection of the local hydrogen-bonding environment. Asparagine and glutamine side-chain orientations were optimized with MolProbity, (version 4.5.8) and the resulting structures were energy-minimized for 1000 steps under the AMBER ff14SB force field with positional restraints (10 kcal mol^−1^ Å^−2^) on all heavy atoms to relieve steric clashes introduced by hydrogen placement.

The twenty-six LC/MS annotated metabolites were retrieved from the PubChem database in SDF format, converted to 3D structures with Open Babel (v3.1.1), and energy-minimized at the MMFF94 level (1000 steepest-descent steps followed by conjugate-gradient convergence to a gradient threshold of 0.01 kcal mol^−1^ Å^−1^). Ionizable groups were assigned protonation states corresponding to physiological pH (7.4), and a single low-energy conformer was retained for each ligand. The two co-crystallized inhibitors (celecoxib and rofecoxib) were extracted from their parent crystal structures, processed identically, and used as redocking controls. Protein and ligand structures were converted to AutoDock PDBQT format with AutoDockTools (v1.5.7), with Gasteiger charges assigned and rotatable bonds defined automatically.

### 2.8. Molecular Docking

Docking calculations were performed with AutoDock Vina (v1.2.5) [26] using a grid box of 22 × 22 × 22 Å centered on the centroid of the co-crystallized inhibitor in each parent structure, a spacing that ensures full coverage of the cyclooxygenase channel including the secondary side-pocket characteristic of COX-2. The exhaustiveness parameter was set to 32, the number of binding modes to 10, and the energy range to 4 kcal mol^−1^; all other parameters were retained at their default values. The docking protocol was first validated by re-docking the co-crystallized ligands (celecoxib in COX-1 and rofecoxib in COX-2) into their parent binding sites, and pose fidelity was assessed by computing the heavy-atom RMSD between the top-ranked docked pose and the experimental crystallographic conformation. Re-docking RMSDs of 1.41 Å (COX-1/celecoxib) and 1.34 Å (COX-2/rofecoxib) are both well within the 2.0 Å threshold conventionally accepted as evidence of pose fidelity, which confirmed the validity of the protocol prior to virtual screening of the metabolite library. The twenty-six P3 metabolites were then docked sequentially into both isoforms, and the top-ranked pose of each ligand was retained for subsequent analysis. The docking score reported throughout this manuscript corresponds to the AutoDock Vina-derived value associated with the top-ranked pose.

Two-dimensional protein–ligand interaction maps were generated for the top-ranked docked poses of all 26 metabolites against both isoforms using Pymol 3.1.0. Visualizer [27].

### 2.9. Molecular Dynamics Simulations

All-atom MD simulations were performed for eight systems in total: the apo (free) form of each isoform, the co-crystallized inhibitor complex of each isoform (celecoxib/COX-1 and rofecoxib/COX-2), and the apigenin and kaempferol complexes of each isoform. The starting structures of the ligand-bound systems were the top-ranked Vina docking poses. Simulations were conducted with GROMACS [28] using the AMBER ff14SB force field [29] for the protein and the General Amber Force Field (GAFF2) for the ligands, with ligand atomic charges derived under the restrained electrostatic potential (RESP) protocol at the HF/6-31G(d) level using Gaussian and the antechamber utility of AmberTools23, (AmberTools23).

Each protein–ligand complex was solvated in a cubic simulation box with a minimum solute–wall distance of 12 Å, using the TIP3P water model. The systems were neutralized by addition of Na^+^ or Cl^−^ counterions as appropriate, and physiological ionic strength (0.15 M NaCl) was achieved by addition of further Na^+^/Cl^−^ pairs. Energy minimization was carried out in two stages: a steepest-descent minimization (5000 steps) with positional restraints (1000 kJ mol^−1^ nm^−2^) on the protein and ligand heavy atoms, followed by an unrestrained conjugate-gradient minimization to a maximum force tolerance of 100 kJ mol^−1^ nm^−1^.

Equilibration was performed in two successive stages. In the NVT phase, the system was heated from 0 to 310 K over 200 ps with a velocity-rescaling thermostat (τ = 0.1 ps) under positional restraints on the protein and ligand heavy atoms. The NPT phase comprised a 500 ps simulation at 310 K and 1 atm, using the Parrinello–Rahman barostat (τ = 2.0 ps, isothermal compressibility 4.5 × 10^−5^ bar^−1^) with the same positional restraints, allowing the solvent density to equilibrate to its physiological value.

Production MD was performed in the NPT ensemble at 310 K and 1 atm for 200 ns per system, with all positional restraints removed. The leapfrog integrator was used with a 2 fs time step, with bonds involving hydrogen atoms constrained by the LINCS algorithm. Long-range electrostatic interactions were treated with the particle-mesh Ewald (PME) method using a real-space cut-off of 12 Å and a Fourier grid spacing of 1.6 Å, and Lennard–Jones interactions were truncated at the same 12 Å cut-off with a force-switching function applied between 10 and 12 Å. Coordinates were written to the trajectory every 100 ps, yielding 2000 frames per 200 ns simulation.

### 2.10. Trajectory Analysis

All trajectory analyses were performed on the production segments of the simulations using the GROMACS analysis tools, MDAnalysis. Backbone Cα-RMSD was computed using the energy-minimized starting structure as reference after rigid-body alignment on the Cα atoms. Ligand heavy-atom RMSD was computed against the t = 0 ns docked pose after first aligning each frame on the protein backbone, ensuring that the reported value reflects ligand displacement within the binding site rather than global protein motion. The radius of gyration (Rg) of the Cα backbone was computed frame-by-frame as a measure of global fold compactness. All time-series quantities were smoothed with a 5 ns rolling window for visualization and reported as mean ± standard deviation computed over the equilibrated portion of each trajectory (typically t = 50–200 ns; the equilibration cut-off was determined by visual inspection of the Cα-RMSD curve for each system).

Principal component analysis (PCA) was performed on the Cartesian coordinates of the Cα atoms after removal of overall translation and rotation by alignment on the equilibrated reference structure. The covariance matrix was diagonalized, and the trajectory was projected onto the first two principal components (PC1 and PC2), which together captured 90–100% of the variance retained on the first two modes for all systems. Free-energy landscapes (FELs) were constructed by Boltzmann inversion of the joint PC1/PC2 probability density according to the following equation:

Δ*G*(PC1, PC2) = −k_β_ T ln [P(PC1, PC2)/P_max_]

where:

Δ*G*: change in Gibbs free energy.
PC1, PC2: the reaction coordinates (principal components).
k_β_: Boltzmann constant.
T: temperature in Kelvin.
ln: natural logarithm.
P(PC1, PC2): the probability density of a specific state.
P_max_: the maximum probability density in the distribution (the global minimum).

The FELs were rendered with a shared color scale (0 to 4.75 kcal mol^−1^) across all systems within each isoform to permit direct visual comparison of basin depth and connectivity.

## 3. Results

### 3.1. The IC_50_ Values of DPPH Radical-Scavenging Activity

The DPPH assay was employed to evaluate the free radical-scavenging capacity of the propolis extracts, using ascorbic acid as a positive control. In this assay, the presence of antioxidants in the propolis samples leads to a reduction of the DPPH radical, which results in a color change from purple-blue to yellow as the radical reacts with hydrogen-donating antioxidants. The extent of this decolorization, which reflects the scavenging activity, was measured using a UV/VIS spectrophotometer. A lower absorbance indicates a greater scavenging effect, as it reflects a higher capacity of propolis extracts to neutralize free radicals.

To evaluate the antioxidant potential of the tested samples (P1, P2, P3 and P4) using DPPH radical-scavenging activity, the IC_50_ values were calculated, representing the concentration required to scavenge 50% of the free radicals. Samples with lower IC_50_ values exhibit greater antioxidant activity. All propolis extracts were estimated using ascorbic acid as positive control. Among the tested samples, the **P3** extract exhibited the highest antioxidant-scavenging activity, surpassing that of ascorbic acid, with an IC_50_ value of 25.84 ± 0.96 µg/mL compared to 27.45 ± 1.42 µg/mL for ascorbic acid. In contrast, the **P1** extract exhibited the lowest antioxidant activity, with an IC_50_ value of 84.16 ± 3.13 µg/mL (Table 1).

### 3.2. ABTS Antioxidant Assay

The ABTS tests the antioxidant activity by detecting the ability of antioxidants to stabilize free radicals, which causes color fading. ABTS is a blue-green radical centered on nitrogen. When antioxidants diminish ABTS, they convert to a non-radical form that changes color from colorful to colorless. The ABTS technique is highly susceptible to light. Antioxidants’ ability to decrease ABTS was assessed using spectrophotometry at 734 nm.

According to Table 1, the IC_50_ data values obtained from antioxidant testing using the ABTS method on four propolis extracts (P1, P2, P3 and P4) were 121.84 ± 4.53, 109.22 ± 3.46, 32.30 ± 1.2 and 79.05 ± 2.94 in µg/mL, respectively. ABTS radical cation decolorization was carried out to confirm the antioxidant activity of the extracts. The results showed that the ABTS free radical-scavenging activity is similar to the activity of DPPH assay. The **P3** extract exhibited the highest antioxidant activity with IC_50_ = 32.30 ± 1.2 µg/mL compared to ascorbic acid IC_50_ = 21.22 ± 0.79 µg/mL, while **P1** displayed the lowest activity, with IC_50_ = 121.84 ± 4.53 µg/mL.

### 3.3. COX-1 and COX-2 Anti-Inflammatory Activities

Cyclooxygenase (COX), also known as prostaglandin H_2_ synthase, is the primary enzymatic target of most non-steroidal anti-inflammatory drugs (NSAIDs). COX-1 is the constitutive isoform of the enzyme that is responsible for the synthesis of prostaglandins involved in maintaining physiological homeostasis, such as gastric and renal functions. In contrast, COX-2 is an inducible isoform that is upregulated in response to cytokines and growth factors, and its expression is predominantly associated with inflammatory cells and tissues [30]. Current pharmaceutical research focuses on the development of selective COX-2 inhibitors, as these agents are expected to provide anti-inflammatory benefits with reduced gastrointestinal and renal side effects compared to non-selective NSAIDs [31].

The COX inhibition assays were conducted using celecoxib as a positive control, which showed IC_50_ values of 24.56 ± 0.83 µg/mL and 0.681 ± 0.02 µg/mL for COX-1 and COX-2, respectively. Among the tested extracts, **P1** exhibited the most potent COX-1 inhibition, with an IC_50_ value of 7.733 ± 0.26 µg/mL, exceeding the efficacy of celecoxib against the COX-1 enzyme. **P3** also demonstrated activity close to that of celecoxib, with an IC_50_ of 23.6 ± 0.79 µg/mL. On the other hand, **P4** showed the weakest COX-1 inhibitory effect (IC_50_ = 42.89 ± 1.44 µg/mL).

Regarding COX-2 inhibition, **P3** exhibited the most significant activity, with an IC_50_ of 6.194 ± 0.21 µg/mL, followed by **P4** (IC_50_ = 15.23 ± 0.51 µg/mL), whereas **P2** showed the least COX-2 inhibition (IC_50_ = 25.78 ± 0.87 µg/mL) (Table 2).

According to Ang & Uy (2023) [32], samples with a COX-1/COX-2 ratio ≤ 1.00 are considered COX-1-selective inhibitors, while those with a ratio ≥ 1.00 are considered COX-2-selective inhibitors. Based on this classification, **P1** was the only extract demonstrating COX-1 selectivity, whereas **P2, P3, P4,** and the reference drug celecoxib were all classified as COX-2-selective inhibitors, with the **P3** extract revealed the most active extract among the tested extracts as a COX-2-selective inhibitor agent.

### 3.4. LC/MS Chemical Profiling

**Figure 1:**
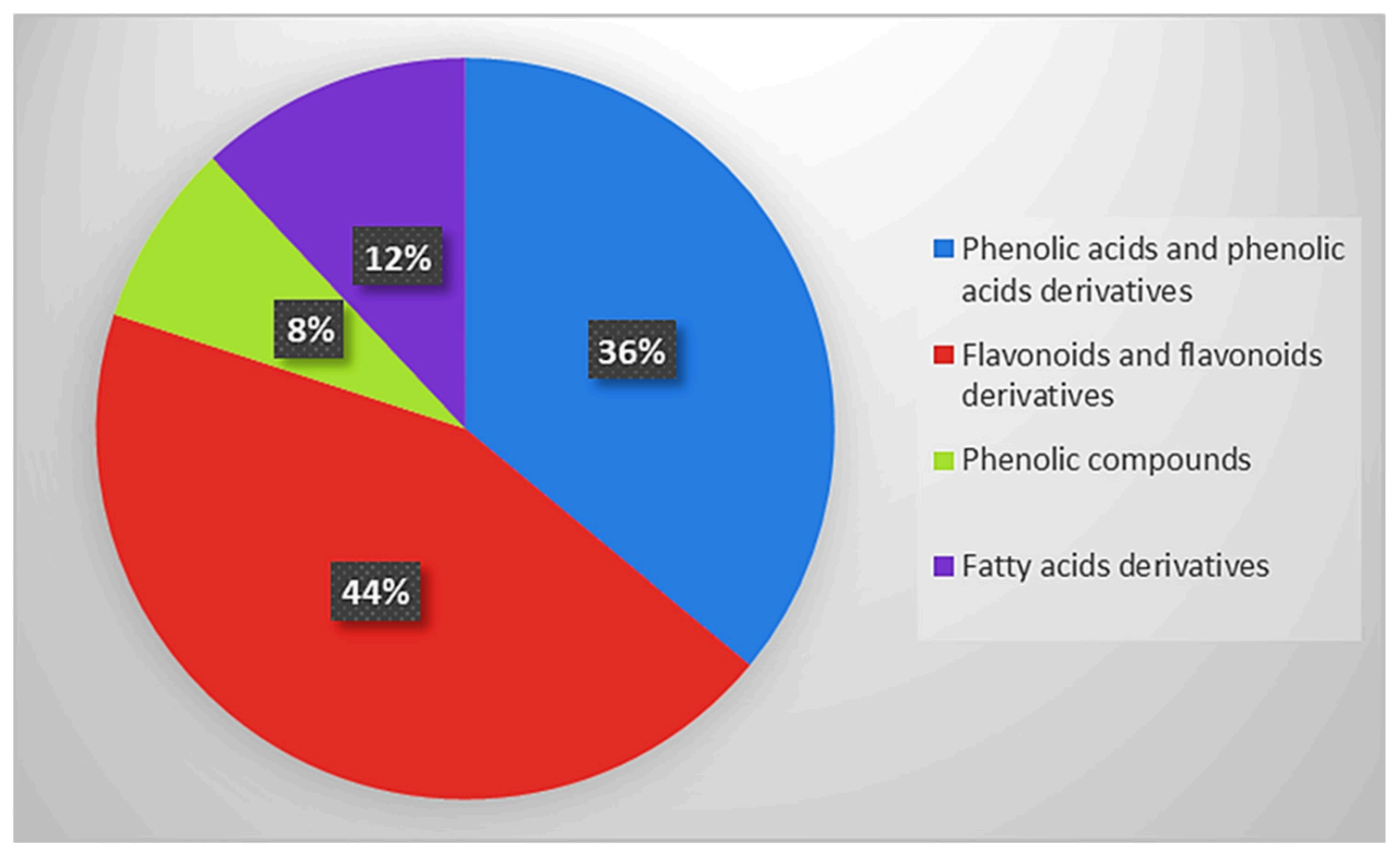
The percentage of the chemical class dereplicated by the LC/MS technique.

The **P3** sample represented the most active and promising sample among all samples under study; so, the need to identify its chemical profile is required. The **P3** sample was subjected to further analysis using LC/MS in positive and negative modes. Only low-molecular-weight (*m*/*z* < 1500) ionizable molecules were taken into consideration when profiling the metabolites using docking studies. DE replication was carried out using the DNP database. The number of obtained characteristics was then decreased by applying a chemotaxonomic filter, leading to the annotation of twenty-six metabolites (Table 3, Figure 1) from different classes, in which flavonoids and flavonoid derivatives were found to prevail among the detected metabolites classes, followed by phenolic acids and phenolic acids derivatives.

### 3.5. Molecular Docking Studies

The reliability of the docking protocol was first established by re-docking the co-crystallized ligands of the two target structures into their parent binding sites. For COX-1 (PDB ID: 3KK6), celecoxib was reproduced with a docking score of −8.1 kcal/mol and a heavy-atom RMSD of 1.41 Å relative to the crystallographic conformation. For COX-2 (PDB ID: 5KIR), rofecoxib was reproduced with a score of −10.4 kcal/mol and an RMSD of 1.34 Å. Both RMSD values fall within the 2.0 Å threshold accepted as evidence of pose fidelity, and the recovered poses preserved the canonical interactions reported for each isoform: the salt-bridge and hydrogen-bonding network engaged by the celecoxib sulfonamide moiety with Arg120 and Tyr355 in COX-1, and the deep insertion of the rofecoxib sulfone group into the secondary pocket bordered by Arg513, His90, and Val523 in COX-2. The protocol was therefore considered suitable for screening the propolis metabolite library [44].

#### 3.5.1. Binding-Site Coverage of the Propolis Metabolites (P3) Against COX-1

The twenty-six metabolites annotated in the LC/MS profile of the **P3** extract were docked into the cyclooxygenase channel of COX-1; the docking scores, together with the principal residue contacts, are summarized in Table 4 Across the library, the scores ranged from −9.2 kcal/mol (apigenin and kaempferol) to −4.9 kcal/mol (fumaric acid). The most favorable binders, namely apigenin (−9.2), kaempferol (−9.2), galangin-5-methyl ether (−8.9), chrysin (−8.8), pinocembrin (−8.8), epicatechin gallate (−8.8), kaempferol methyl ether (−8.7), pinobanksin (−8.4), and miquelianin (−8.4 kcal/mol), all surpassed the redocked celecoxib reference (−8.1 kcal/mol), an observation consistent with the affinity of the flavonoid scaffold for the COX hydrophobic channel. Chemotype dependence was evident: planar flavonoid aglycones occupied the upper tier of scores, glycosylated and gallate-substituted derivatives occupied an intermediate tier, and the small phenolic acids (fumaric acid, protocatechuic aldehyde, nicotiflorin, and dicaffeoylquinic acid) occupied the lowest tier, reflecting their inability to satisfy the predominantly lipophilic contacts demanded by the cyclooxygenase channel.

**Figure 2:**
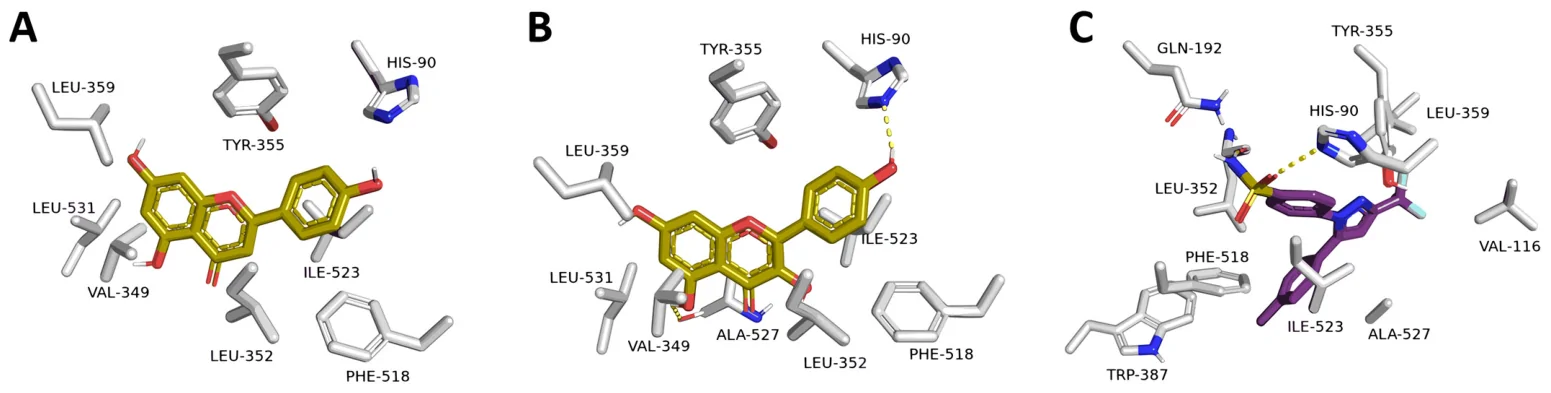
Three-dimensional binding pose of apigenin, kaempferol, and celecoxib (panels (**A**), (**B**), and (**C**), respectively) inside the active site of COX-1 (PDB ID: 3KK6).

Inspection of the top-scoring poses revealed a recurrent binding mode in which the chromone or flavone core inserts deep into the hydrophobic lobby of the channel, sandwiched between Val349, Leu352, Leu531, Tyr355, Phe518, and Trp387, while the B-ring projects toward the constriction defined by Ile523. This residue, the gate-keeper of the COX-1 catalytic channel, was systematically engaged through a π–σ interaction with the flavonoid B-ring in apigenin, kaempferol, chrysin, and pinocembrin. The hydrogen-bonding pattern was comparatively sparse: kaempferol contributed two conventional hydrogen bonds (4′-OH···His90 and 5-OH···Ala527), apigenin formed only weak carbon–hydrogen contacts with Ala527 (Table 4, Figure 2) and Ser530, and chrysin established a single 5-OH···Ser530 hydrogen bond. The tightly packed Ile523 wall in COX-1 therefore restricts the ligands to a planar, lobby-bound pose in which polar contacts are limited to peripheral residues, and the affinity of the top binders is dominated by hydrophobic and π-stacking contributions rather than by directional polar recognition.

#### 3.5.2. Binding-Site Coverage of the Propolis Metabolites Against COX-2

Re-docking the twenty-six metabolites into COX-2 produced a uniformly more favorable scoring distribution, ranging from −10.3 kcal/mol (apigenin) to −4.5 kcal/mol (fumaric acid) (Table 5). The flavonoid aglycones again populated the top tier, with apigenin (−10.3), kaempferol (−10.2), chrysin (−9.2), galangin-5-methyl ether (−9.2), pinocembrin (−9.1), and kaempferol methyl ether (−9.1 kcal/mol), all clustering within ~1.3 kcal/mol of the redocked rofecoxib reference (−10.4 kcal/mol). Apigenin in particular reached a score essentially equivalent to that of a clinically validated selective COX-2 inhibitor, an outcome that is notable for a natural product lacking the sulfonamide or sulfone pharmacophore that ordinarily confers COX-2 selectivity (Table 5).

**Figure 3:**
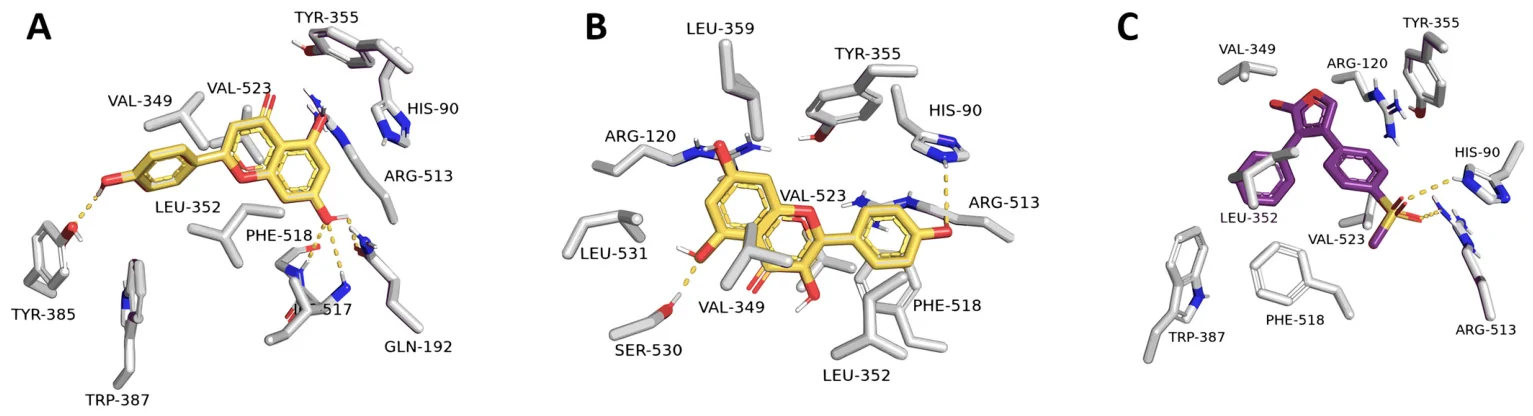
Three-dimensional binding pose of apigenin, kaempferol, and rofecoxib (panels (**A**), (**B**), and (**C**), respectively) inside the active site of COX-2 (PDB ID: 5KIR).

The interaction maps of the high-scoring poses revealed three structural features that account for the more favorable accommodation of these metabolites in COX-2 relative to COX-1. First, recognition of Arg120 at the channel entrance: apigenin, kaempferol, and chrysin each engaged Arg120 through a π–cation interaction with the chromone A-ring; this interaction is geometrically permitted in COX-2 because the wider channel allows the flavonoid to seat itself with the A-ring facing Arg120, whereas the equivalent pose in the narrower COX-1 channel forces the molecule to relinquish this contact. Second, engagement of the gate-keeper residue Val523, which corresponds to the bulkier Ile523 in COX-1 and defines the entry to the secondary side-pocket that constitutes the structural basis of COX-2 selectivity: in COX-2, kaempferol and apigenin (Figure 3) formed π–σ and π–alkyl contacts with Val523, allowing the B-ring to lean toward the side-pocket; the same contact in COX-1 was sterically tighter and unproductive, since Ile523 enforces a planar, lobby-restricted pose. Third, polar anchoring at Ala527 and Ser530: both apigenin and kaempferol formed carbon–hydrogen bonds with Ala527, and kaempferol additionally established a conventional hydrogen bond from its 5-OH to Ala527 (Figure 3). The composite interaction pattern of kaempferol, comprising π–cation (Arg120), π–σ (Val523), conventional H-bond (Ala527), and a π–alkyl array (Val349, Tyr355, Leu531), recapitulates four of the five contacts also formed by the redocked rofecoxib (Arg513, His90, Val523, Val349, Phe518), differing principally in that rofecoxib reaches further into the side-pocket and engages Arg513 and His90 directly, whereas the flavonoid scaffold remains anchored at the channel entrance via Arg120. The redocked rofecoxib pose exemplifies the canonical side-pocket engagement that defines second-generation coxibs and provides a structural reference against which the flavonoid binding mode can be benchmarked.

#### 3.5.3. Comparative Analysis and Selection Rationale

A direct comparison of the docking scores obtained against the two isoforms (Δscore = score-COX-2 − score-COX-1) was used as a structure-based proxy for COX-2 preference, noting that this comparison is internal to the scoring function and does not estimate absolute binding free energy for either isoform. Discrimination between paralogous targets by score differences is subject to the limitations set out in Section 3.5.4 [44], and is not resolved by the subsequent MD analysis, which characterizes conformational stability rather than binding thermodynamics. The most pronounced COX-2 preference was obtained for apigenin (Δscore = −1.1 kcal/mol), followed by kaempferol (−1.0), pinobanksin (−0.5), chrysin (−0.4), kaempferol methyl ether (−0.4), galangin-5-methyl ether (−0.3), and pinocembrin (−0.3 kcal/mol). All members of this short list belong to the flavone or flavanone subfamily and share the unsubstituted A-ring 5-OH and 7-OH pattern, which appears to be the structural determinant that allows for simultaneous engagement of the Arg120 anchor and the Val523 gate-keeper in COX-2. Compounds in which the A-ring is glycosylated (genistin, miquelianin, nicotiflorin) or in which the polyphenolic core is sterically expanded by a galloyl group (epigallocatechin gallate, epicatechin gallate) sacrificed this dual recognition; their isoform preference flattened or, in the case of epicatechin gallate, marginally inverted toward COX-1.

On the combined basis of the (i) absolute COX-2 docking score, (ii) magnitude of COX-2-over-COX-1 preference, (iii) recapitulation of the Arg120/Val523/Ala527 interaction pattern characteristic of selective COX-2 binders, and (iv) congruence with the experimental COX inhibition data in which the P3 extract was the most COX-2-selective sample, apigenin and kaempferol were prioritized as the two lead candidates for the subsequent dynamic characterization. Chrysin and pinobanksin were retained as secondary candidates because they reproduce the same recognition motif at slightly diminished scores and may help establish the pharmacophoric requirements within the flavonoid series. These four compounds were therefore advanced to 200 ns molecular dynamics simulations to evaluate the stability of their COX-2 complexes.

A methodological note on the interpretation of docking scores is warranted. The AutoDock Vina-derived scores reported in Table 5 represent empirical estimates from a knowledge-based scoring function and should not be equated with experimental binding affinities or used as quantitative predictors of biological IC_50_ values. The scores are employed here in their appropriate role: rank-ordering compounds within a structurally related series to guide the selection of candidates for the more computationally rigorous MD phase. Furthermore, the docking grid was centered on the co-crystallized inhibitor binding site in each structure, encompassing the full cyclooxygenase channel and the secondary side-pocket. No allosteric site with established functional relevance to COX-1 or COX-2 inhibition is documented in the literature; this study was therefore designed exclusively around competitive binding at the catalytic channel, which is consistent with the mechanism of all clinically approved COX inhibitors.

#### 3.5.4. Interpretation and Limitations of the Docking Scores

The values in Table 5 require careful interpretation. The free energy of a protein–ligand association is the difference between the free energy of the complex and the summed free energies of the free protein and the free ligand in solution:

Δ*G*(binding) = *G*(complex) − [*G*(free protein) + *G*(free ligand)]

Empirical scoring functions, including that of AutoDock Vina, evaluate the assembled complex and therefore address the first term only. The free energies of the desolvated protein and of the ligand in bulk solvent are not computed and remain unknown. A favorable score consequently does not establish binding: a ligand with greater affinity for the solvent than for the cyclooxygenase channel will remain predominantly in the solution regardless of how favorably the complex scores, and may be a nonbinder despite ranking highly. End-point methods such as MM-PBSA and MM-GBSA are subject to the same restriction, compounded by continuum solvation models, the neglect or crude approximation of configurational entropy, and sensitivity to dielectric and force-field parameterization, which together introduce errors of unknown magnitude and significance for any individual system. Neither docking nor MM-PBSA/GBSA yields an absolute binding free energy. At best, they rank ligands against a common target in the correct order, and they fail at this with sufficient frequency that the ranking itself requires independent validation [44].

The protocol used here was validated by re-docking the co-crystallized inhibitors, which reproduced their crystallographic poses to within 1.41 Å (COX-1) and 1.34 Å (COX-2). This establishes pose fidelity and nothing further. Re-docking a ligand that is known to bind confirms that the scoring function can identify the correct geometry when the correct answer already exists in the target structure; it does not test whether the function assigns unfavorable scores to compounds that do not bind. A protocol that scores all submitted compounds favorably will generate a plausible ranking of any library while conveying no information, and positive controls alone cannot detect this failure mode.

### 3.6. Molecular Dynamics Simulations

Apigenin and kaempferol were selected for MD simulation on the basis of four criteria applied in Section 3.5.3: highest absolute COX-2 docking scores in the library (−10.3 and −10.2 kcal/mol, respectively), largest COX-2-over-COX-1 score differentials (−1.1 and −1.0 kcal/mol), full recapitulation of the Arg120/Val523/Ala527 contact triad that defines selective COX-2 recognition, and congruence with the experimental finding that P3 is the most COX-2-selective extract. The co-crystallized inhibitors of each isoform (celecoxib for COX-1, rofecoxib for COX-2) were included as dynamic references, and the apo forms of both enzymes were simulated to establish the unliganded baseline.

To probe the dynamic behavior and conformational stability of the protein–ligand complexes identified in the docking step, 200 ns all-atom MD simulations were performed for the apo enzyme and for the apigenin, kaempferol, and co-crystallized inhibitor complexes of both COX-1 and COX-2 isoforms. The four metrics interrogated below, namely backbone Cα-RMSD, ligand heavy-atom RMSD, radius of gyration (Rg), and PCA-based free-energy landscapes, collectively report on the global structural stability, persistence of the bound pose, compactness of the tertiary fold, and the topology of the conformational subspace explored by each system over the simulation window.

**Figure 4:**
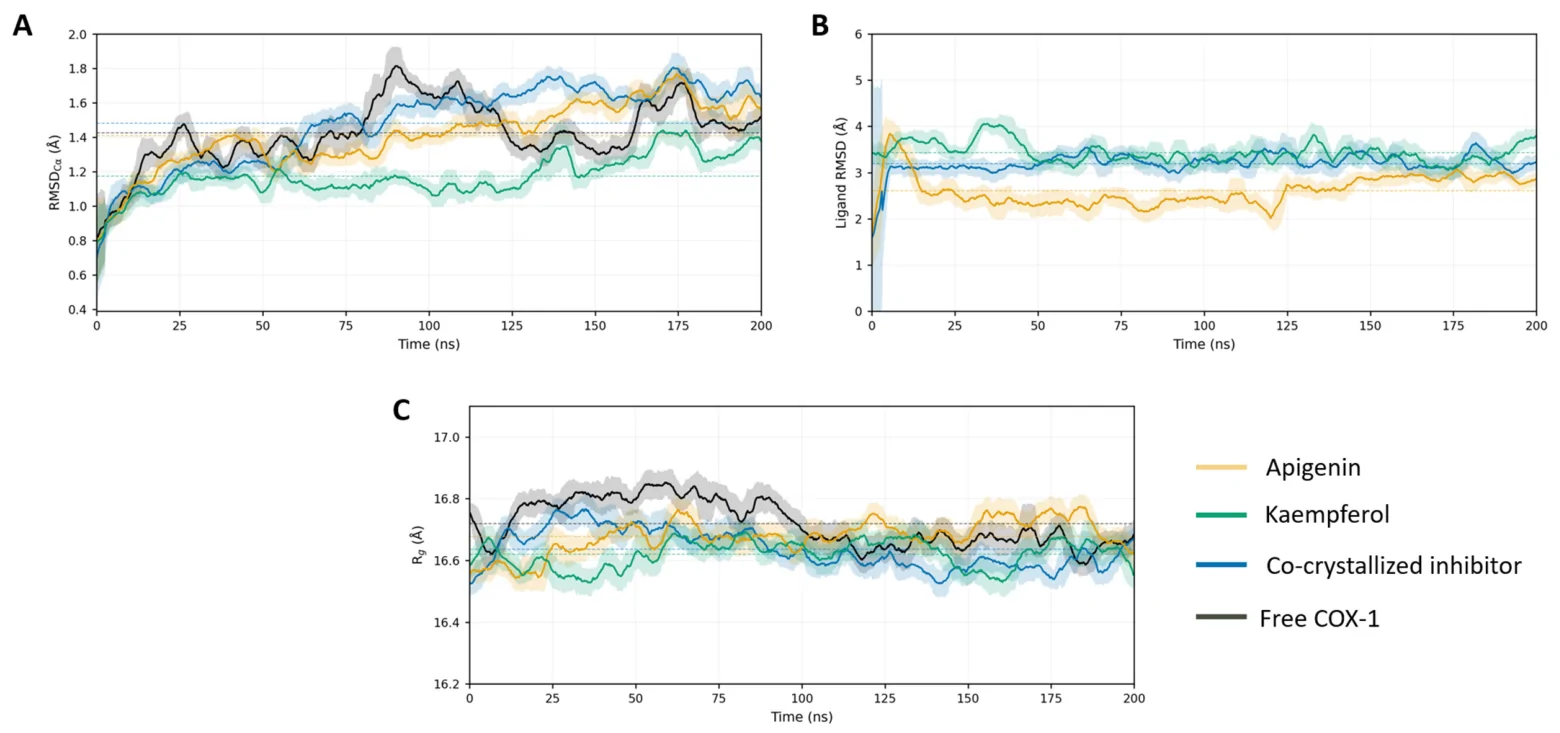
Molecular dynamics simulation analysis of kaempferol, apigenin, and the co-crystallized inhibitor in complex with COX-1 over 200 ns. (**A**) Backbone Cα-RMSD time series for the free enzyme (black), the co-crystallized inhibitor complex (blue), apigenin (orange), and kaempferol (green). Dashed horizontal lines indicate the trajectory mean for each system. (**B**) Ligand heavy-atom RMSD relative to the docked pose. (**C**) Radius of gyration (Rg) of the Cα backbone.

In the COX-1 simulations (Figure 4), all four systems reached a stable plateau within the first 50 ns and remained equilibrated for the remainder of the trajectory. The apo enzyme, the celecoxib complex, and the apigenin complex converged to overlapping mean Cα-RMSD values of 1.43 ± 0.20, 1.48 ± 0.24, and 1.41 ± 0.20 Å, respectively, indicating that ligand occupancy of the COX-1 channel does not substantially perturb the backbone fluctuations relative to the apo state. Kaempferol, by comparison, exhibited a damped backbone profile that stabilized around 1.18 ± 0.14 Å, which is approximately 0.25 Å below the apo baseline and the lowest mean RMSD across all four COX-1 trajectories, indicating that kaempferol binding actively rigidifies the COX-1 backbone rather than merely tolerating the unbound dynamics.

The COX-2 simulations (Figure 4) reproduced the same hierarchy at slightly elevated absolute values, consistent with the intrinsic flexibility of the COX-2 fold. The apo state averaged 1.83 ± 0.39 Å, the rofecoxib complex 1.93 ± 0.38 Å, and the apigenin complex 1.79 ± 0.38 Å. Kaempferol again produced the most stabilized trajectory of the set, with a mean Cα-RMSD of 1.61 ± 0.29 Å and the narrowest standard deviation, reproducing the rigidifying effect already observed in COX-1. The cross-isoform consistency of this rigidification, with kaempferol acting as the strongest backbone stabilizer in both COX-1 and COX-2, argues that the effect originates in a common structural mechanism (likely the dual engagement of the gate-keeper residue and the Arg120 anchor identified in the docking analysis) rather than in an isoform-specific contact pattern.

The persistence of the docked pose was assessed by tracking the heavy-atom RMSD of each ligand against its pose at t = 0 ns after backbone alignment. In the COX-1 channel (Figure 4), the celecoxib reference equilibrated near 3.20 ± 0.50 Å, indicating moderate residual mobility of the bulky sulfonamide scaffold within the cyclooxygenase lobby. Apigenin showed the largest pose drift of the three ligands, fluctuating around 3.43 ± 0.32 Å, while kaempferol settled into a tighter trajectory at 2.61 ± 0.42 Å. The flat, low-amplitude profile of the kaempferol curve from 25 ns onward indicates that the docked pose is dynamically retained, consistent with the additional 4′-OH···His90 and 5-OH···Ala527 hydrogen bonds resolved in the docking analysis; these directional polar contacts coincide with the reduced pose fluctuation observed for kaempferol relative to apigenin over the sampled window. No inference about dissociation kinetics is drawn since 200 ns is orders of magnitude shorter than the timescale on which such complexes dissociate.

**Figure 5:**
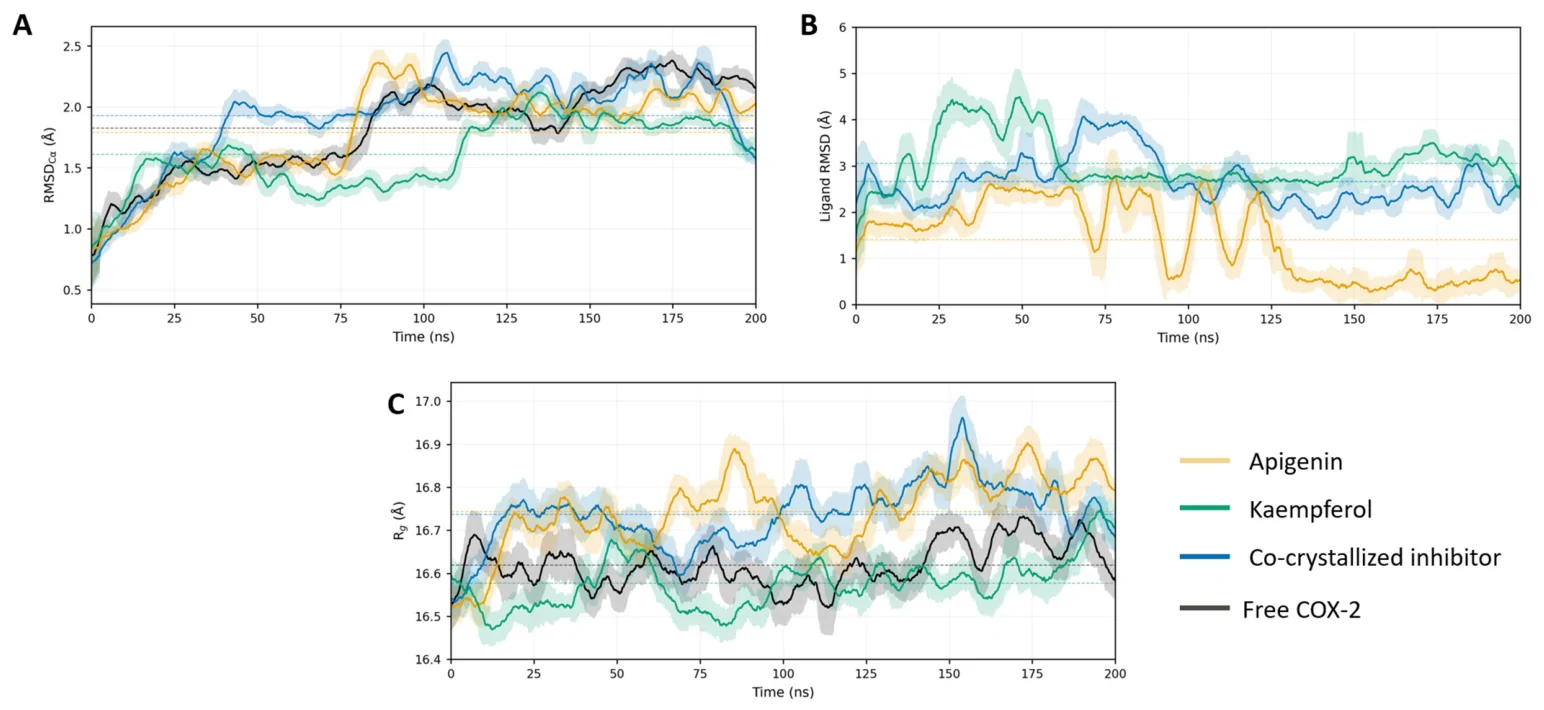
Molecular dynamics simulation analysis of kaempferol, apigenin, and the co-crystallized inhibitor in complex with COX-2 over 200 ns. (**A**) Backbone Cα-RMSD time series for the free enzyme (black), the co-crystallized inhibitor complex (blue), apigenin (orange), and kaempferol (green). Dashed horizontal lines indicate the trajectory mean for each system. (**B**) Ligand heavy-atom RMSD relative to the docked pose. (**C**) Radius of gyration (Rg) of the Cα backbone.

The COX-2 trajectories (Figure 5) revealed a different and biologically important pattern. Rofecoxib was used as the reference ligand for the COX-2 simulations (consistent with the docking phase), and the rofecoxib complex equilibrated at 2.66 ± 0.62 Å, a value typical of a clinically validated COX-2-selective inhibitor occupying its native binding mode. Apigenin again exhibited the highest pose drift of the three ligands at 3.06 ± 0.67 Å, with the trajectory remaining elevated above the rofecoxib baseline throughout the simulation. Kaempferol, in contrast, achieved a tightened pose with a mean RMSD of 1.41 ± 0.91 Å, lower than both the rofecoxib reference and its own performance in COX-1, and lower than the t = 0 ns docked geometry by the second half of the trajectory. Beyond the mean value, the kaempferol RMSD profile drops progressively from ~2 Å at the start to <1 Å after 125 ns, indicating an induced-fit relaxation in which the ligand reorganizes into a deeper, more stable binding mode rather than simply maintaining the docked pose. The wider standard deviation (±0.91 Å) reflects the transition between the two metastable poses rather than instability of the bound state.

The radius of gyration (Rg) of the Cα backbone was used as an orthogonal indicator of global fold compactness. In the COX-1 simulations (Figure 5), the four systems oscillated within a narrow 0.10 Å envelope around 16.6–16.7 Å throughout the trajectory: apo at 16.72 ± 0.09, celecoxib at 16.64 ± 0.08, apigenin at 16.68 ± 0.07, and kaempferol at 16.62 ± 0.06 Å. The differences are at the limit of numerical resolution and indicate that ligand binding does not measurably perturb the overall packing of the COX-1 globular fold, although kaempferol again returned the tightest distribution, consistent with its damped Cα-RMSD profile.

The COX-2 Rg traces (Figure 5) showed a more clearly resolved divergence between systems. The apo enzyme equilibrated at 16.62 ± 0.07 Å and kaempferol at 16.58 ± 0.07 Å, both compact and effectively indistinguishable, whereas the rofecoxib (16.74 ± 0.09 Å) and apigenin (16.74 ± 0.10 Å) complexes drifted upward to a measurably expanded conformation, with the apigenin trajectory reaching transient maxima above 16.85 Å in the second half of the simulation. The directionality of this difference is consistent with the ligand RMSD analysis: kaempferol settles into a tightly packed, compact COX-2 fold, while apigenin maintains a more dilated channel geometry, presumably reflecting an unrelaxed pose in which the ligand fails to engage the deeper sub-pocket contacts available to a properly accommodated COX-2 binder. The fact that the kaempferol-bound COX-2 fold is as compact as the apo state, despite an additional ~270 Da of ligand mass occupying the active site, indicates that kaempferol binding is fully accommodated by the native fold without requiring channel expansion, a feature characteristic of an energetically well-matched protein–ligand complex.

**Figure 6:**
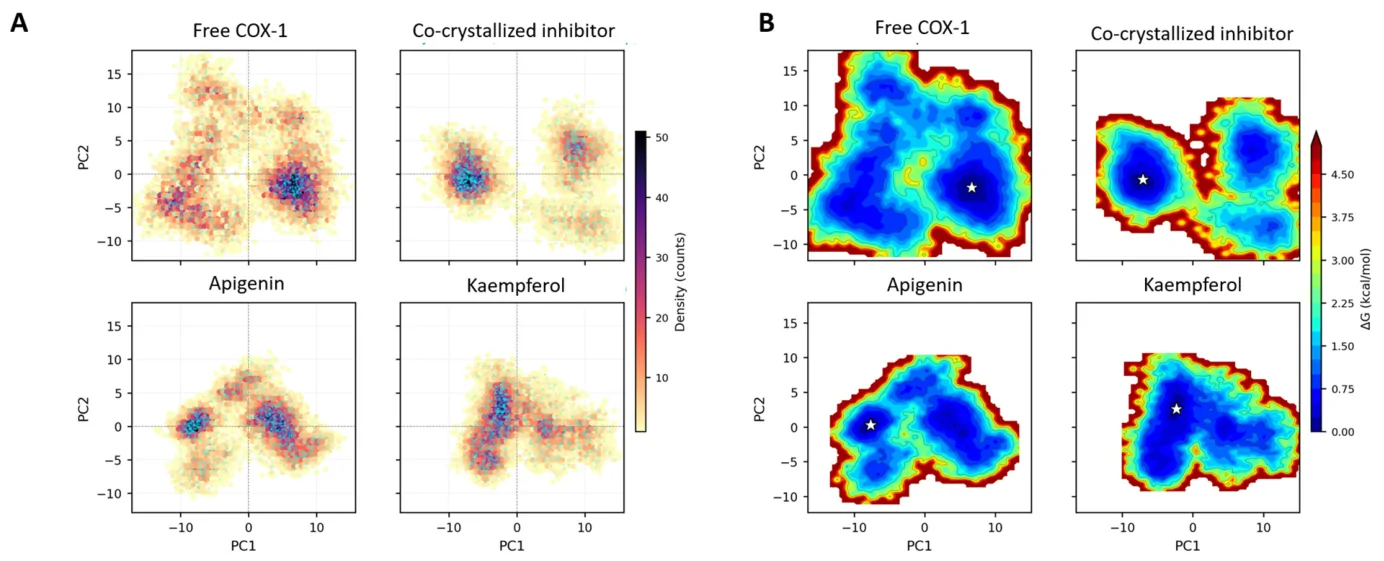
Principal component analysis and free-energy landscape of COX-1 MD trajectories. (**A**) PC1 versus PC2 conformational scatter plots. (**B**) Free-energy landscapes (FELs) constructed by Boltzmann inversion of the PC1/PC2 probability density (Δ*G* = −k_β_T·ln[P(PC1, PC2)/P_max_]; kcal/mol, shared color scale 0 to 4.75).

PCA was performed on the Cα coordinates of each trajectory to project the 200 ns conformational ensemble onto the two dominant collective motions (PC1 and PC2). For COX-1 (Figure 6), PC1 + PC2 captured 100% of the variance retained on the first two components, with PC1 contributing 63.4% in the apo state and rising to 81.4%, 73.5%, and 65.7% in the celecoxib, apigenin, and kaempferol complexes, respectively. The ligand-induced concentration of variance onto PC1 indicates that ligand binding restricts the accessible conformational subspace, channeling the dynamics into a reduced number of dominant modes; the effect is most pronounced for celecoxib and progressively smaller for apigenin and kaempferol. For COX-2 (Figure 6), the same trend was reproduced and intensified: PC1 carried 76.3% of the variance in the apo state and rose to 86.2% in the kaempferol complex (the highest value in the entire dataset), with apigenin (78.7%) and rofecoxib (66.2%) intermediate. The high PC1 dominance in the COX-2/kaempferol simulation (86.2%, with PC2 reduced to 13.8%) indicates that this trajectory is effectively confined to a single dominant collective motion, the dynamic counterpart of a kinetically trapped, well-equilibrated complex.

**Figure 7:**
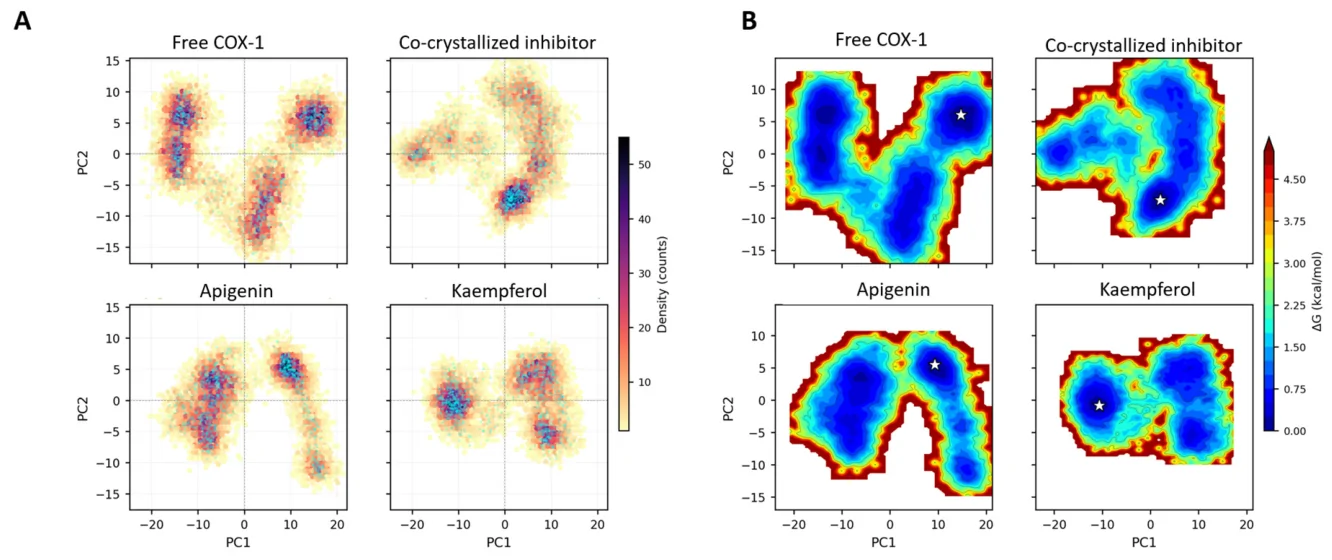
Principal component analysis and free-energy landscape of COX-2 MD trajectories. (**A**) PC1 versus PC2 conformational scatter plots. (**B**) Free-energy landscapes constructed by Boltzmann inversion of the PC1/PC2 probability density.

Free-energy landscapes (FELs) were constructed by Boltzmann inversion of the joint PC1/PC2 probability densities for the four COX-1 systems. The free COX-1 FEL (Figure 7) is broadly distributed and presents two energetically comparable low-energy regions separated by an intermediate ridge of approximately 2 kcal/mol, indicating that the unliganded COX-1 enzyme samples two interconverting conformational states under the simulation conditions. The celecoxib complex consolidates the bulk of the population into a single primary basin located in the negative-PC1 and negative-PC2 quadrant, with a residual secondary minimum reflecting the partial mobility of the celecoxib sulfonamide arm within the cyclooxygenase lobby. The kaempferol/COX-1 FEL is the most clearly funneled of the four COX-1 landscapes: a single deep, narrow basin (Δ*G* = 0 at the global minimum) is surrounded by steep barriers that rise above 3 kcal/mol within a short PC1 displacement, the topological feature of a kinetically committed complex in which the system is dynamically locked into a single representative bound state. The apigenin/COX-1 FEL also resolves a primary minimum, but the basin is broader and shallower than that of kaempferol and is connected to a secondary low-energy region by a sub-kcal/mol barrier, indicating that apigenin tolerates a broader conformational ensemble of bound states.

The FEL analysis was repeated for the four COX-2 systems (Figure 7) and revealed both a similar ligand-induced funneling pattern and an isoform-specific intensification of the kaempferol effect. The apo COX-2 FEL is multi-basin, with three resolved low-energy minima (Δ*G *≈ 0–0.75 kcal/mol) distributed across the negative-PC1, central, and positive-PC1 regions of the projection, separated by barriers of 1.5–2.5 kcal/mol; this topology indicates a more rugged conformational landscape than that of the COX-1 apo equivalent. The rofecoxib complex shows a similarly distributed but slightly smoothened landscape, with its global minimum displaced to the positive-PC1 and negative-PC2 quadrants. The apigenin landscape retains a multi-basin architecture in which two competing minima of comparable depth coexist along the PC1 axis, separated by a shallow 1–1.5 kcal/mol barrier, a topology indicating that apigenin fails to commit the system to a single conformational state and instead allows the trajectory to oscillate between alternative bound geometries, which aligns with its elevated and noisy ligand RMSD profile. The kaempferol landscape, by comparison, is dominated by a single deep, well-defined minimum at PC1 ≈ −10 and PC2 ≈ 0, with the surrounding basin extending into a connected low-energy region; secondary minima are present but shallower and lie within ~1 kcal/mol of the global minimum. This funneled topology, with a single deep basin acting as a dynamical attractor, is consistent with a conformationally converged complex occupying a single dominant basin of the projected landscape. The free-energy values plotted here derive from Boltzmann inversion of the PC1/PC2 population density of the bound complex and describe the relative population of conformational states within it. They are not binding free energies, in agreement with the progressive RMSD relaxation observed in the kaempferol/COX-2 time series. The cross-isoform comparison is informative: the kaempferol FEL is funneled in both COX-1 and COX-2, but the funnel is reached from a more rugged starting landscape in COX-2 (three apo basins versus two in COX-1), indicating that kaempferol does more conformational work to commit the COX-2 enzyme to a single state, the thermodynamic counterpart of the induced-fit relaxation visible in the ligand RMSD trajectory.

Taken together, the four MD descriptors deliver a coherent and isoform-discriminating picture. In COX-1, both flavonoids bind stably but differ in their dynamic signatures: kaempferol exerts a measurable rigidifying effect on the backbone and maintains a tighter pose, while apigenin behaves comparably to the celecoxib reference. In COX-2 the dynamic separation between the two flavonoids becomes pronounced: kaempferol simultaneously achieves the lowest Cα-RMSD, the most compact Rg, the tightest ligand pose (with progressive induced-fit deepening), the highest PC1 variance concentration, and a deep single-basin FEL. Taken together, these four MD descriptors consistently rank the kaempferol/COX-2 complex as the most conformationally stable system among the four COX-2 simulations, including the rofecoxib reference. This finding represents a computational prediction of binding stability and should not be interpreted as experimental evidence of inhibitory potency. Validation through bioassay of the isolated compound, fractionation studies, and direct comparison with the crude extract activity will be required to establish whether kaempferol is a principal contributor to the observed COX-2 inhibition of P3. Despite a marginally higher static docking score (0.1 kcal/mol, within the resolution of the scoring function), apigenin does not display the same dynamic stability and instead shows a partially mismatched binder profile in COX-2.

The sampling window imposes limits on what these trajectories establish. Ligand dissociation from a protein binding site occurs on timescales ranging from milliseconds to seconds for ligands with *k*_off_ values between roughly 100 and 0.2 s^−1^, which is four to seven orders of magnitude beyond the 200 ns simulated here. No dissociation event was observed in any of the six ligand-bound systems, and none would be expected regardless of whether the complexes are experimentally stable. Simulations of comparable protein–ligand and protein–peptide systems over similar windows show complexes remaining fully associated, with even weakly bound or nonbinding pairs displaying at most the initial stages of separation [44]. Sustained association over 200 ns is therefore uninformative regarding true binding, and the comparisons presented above rest on differences in fluctuation amplitude within the bound state rather than on any separation between bound and unbound behavior. These simulations additionally inherit the deficiencies of the underlying force fields, which are known to be over-stabilizing: simulated radii of gyration are compressed relative to experiment and simulated dissociation is slower than measured. This bias acts in the direction of the trends reported here. Behavior observed in a simulation does not establish that the same behavior occurs in vitro or in vivo, and the results above are presented as a relative comparison among identically treated systems rather than as a prediction of experimental stability.

## 4. Discussion

In recent years, propolis has attracted considerable interest for its potential applications in the pharmaceutical industry. The valuable properties of propolis are mainly attributed to its biologically active chemical constituents, which are deeply influenced by geographical origin and botanical diversity [2,45]. Honeybees synthesize propolis by gathering resinous exudates and plant waxes from regional flora; thus, the local vegetation, altitude, and climatic conditions of each zone cause distinct variations in secondary metabolites [2,45].

The therapeutic superiority of P3 extract is intrinsically linked to geographical and climatic parameters of Fayfa Mountain. The mountain rises over 2000 m above sea level, generating a unique humid subtropical climate characterized by high rainfall and moderate temperatures. This environmental matrix hosts 537 vascular plant species, dominated primarily by Fabaceae, Asteraceae, and Afromontane trees like Olea europaea and Juniperus procera. Furthermore, Fayfa’s consistent precipitation prevents seasonal vegetation droughts, allowing honeybees to harvest highly active, unoxidized phenolic resins. In contrast, in other regions like Riyadh (P1) or Al-Madinah (P2), prolonged droughts force honeybees to harvest non-reactive, heavy aliphatic plant waxes to physically protect the hive from dehydration and extreme heat [46,47,48,49].

Phenolic compounds in propolis, particularly those containing a catechol group, are known to neutralize free radicals, while flavonoids act as scavengers during lipid peroxidation, stopping the oxidative damage. The P3 propolis extract demonstrated potent antioxidant activity, as evidenced by its low IC_50_ value in the DPPH assay (25.84 ± 0.96 μg/mL), outperforming or matching the pure ascorbic acid reference standard (27.45 ± 1.42 μg/mL for DPPH). This result was highly consistent with the ABTS assay findings, where P3 also exhibited the strongest antioxidant effect with IC_50_ = 32.30 ± 1.20 μg/mL.

The exceptional radical-clearing capacity and highly efficient enzyme inhibition demonstrated by the P3 extract are directly justified by LC-MS/MS analysis rather than relying on a single compound; P3 revealed 26 annotated secondary metabolites belonging predominantly to phenolic acid and flavonoid classes.

The extract revealed the presence of chlorogenic acid (8.07% vol.), supported by caffeic acid (0.49% vol.) and dicaffeoylquinic acid (0.45% vol.). Structurally, chlorogenic and caffeic acids possess an unhindered catecholic (ortho-dihydroxy) configuration on their phenyl rings. This specific geometry allows for the execution of rapid hydrogen atom transfer (HAT) and sequential proton-loss electron transfer (SPLET) mechanisms. When reacting with DPPH or ABTS radicals, they easily donate a hydrogen atom or electron, forming highly resonance-stabilized phenoxyl radicals that instantly terminate free-radical chain reactions [50,51].

This botanical density is further enriched by distribution of flavonoids, led by miquelianin (quercetin 3-O-glucuronide, 3.43% vol.), naringenin (2.0% vol.), pinobanksin (1.36% vol.), and pinocembrin (1.11% vol.). Miquelianin (a glucuronide conjugate of quercetin) exhibits excellent localized bioavailability and water solubility. Quercetin derivatives are classical multi-target anti-inflammatory agents known to suppress the expression of inducible nitric oxide synthase (iNOS). Furthermore, the combination of pinocembrin and pinobanksin represents a significant lipophilic chemical shield that easily passes through cellular lipid bilayers. Once inside, they act in structural synergy with the water-soluble chlorogenic acid, providing an amphiphilic nature to the P3 matrix. This enables the extract to simultaneously neutralize aqueous-phase radicals (via ABTS) and lipid-phase peroxyl radicals, preventing downstream cellular oxidative stress [52,53,54].

Cyclooxygenase exists in two distinct isoforms: cyclooxygenase-1 (COX-1) and cyclooxygenase-2 (COX-2). COX-1 is constitutively involved in producing cytoprotective prostaglandins in the gastrointestinal tract. In contrast, COX-2 is inducible and synthesized in response to pro-inflammatory stimuli, including TNF-α, IL-6, carrageenan, and histamine. COX-2 plays a central role in inflammation and pain [55]. Drugs like aspirin suppresses COX-1 more strongly, blocking cytoprotective PGE_2_ and PGI_2_, which leads to adverse ulcerogenic effects. Therefore, selective COX-2 inhibitors are highly preferred in modern therapeutics due to their safer gastrointestinal profile [16].

Interestingly, our biological findings revealed distinct screening variations among the regional samples. Extract P1 was the only sample exhibiting rigid COX-1 selectivity (IC_50_ = 7.733 μg/mL), whereas the P2, P3, and P4 extracts were all classified as selective COX-2 inhibitors. Notably, the P3 extract exhibited the highest COX-2 inhibitory activity among all tested samples, achieving a potent IC_50_ of 6.194 ± 0.21 μg/mL and a favorable selectivity ratio.

To determine the molecular causes behind these biological test results, all 26 annotated metabolites from the P3 profile were subjected to computer-aided drug design (CADD) programs, including molecular docking and molecular dynamics (MD) simulations, to clarify target–ligand interactions and predict the primary active candidate. Caffeic acid and its derivatives are well-documented competitive inhibitors of COX-2; due to their low molecular weight and compact planar structures, they slip deep into the hydrophobic pocket without facing steric clashes, forming key hydrogen bonds with active-site residues (such as Arg120 and Tyr355) to block arachidonic acid docking. However, our extensive computational trajectory analyses ranked the kaempferol/COX-2 complex as the most stable system among the four COX-2 simulations, including the rofecoxib reference. Despite displaying a marginally higher static docking score (by a negligible 0.1 kcal/mol, which sits well within the baseline resolution threshold of the scoring function), apigenin failed to maintain dynamic stability, exhibiting a partially mismatched binder profile inside the COX-2 pocket over time. On the other hand, inside the COX-1 pocket, apigenin behaved comparably to the celecoxib reference, whereas kaempferol exerted a measurable rigidifying effect on the enzyme’s backbone, maintaining a significantly tighter, locked pose.

All annotated metabolites were subjected to many CADD programs like molecular docking and molecular dynamics simulations to predict which active constituents may have the strongest anti-inflammatory activity among the annotated active constituents. The computational analyses consistently identify kaempferol as forming the most dynamically stable COX-2 complex in the dataset, a result that makes it a rational candidate for further investigation. However, this designation rests entirely on docking scores and MD-derived stability descriptors. No isolated constituent of P3 was tested for COX inhibitory activity in this study, and the biological data in Table 3 reflect the activity of the whole extract. Kaempferol was also detected at only 0.02% relative abundance in the LC-MS profile, indicating that its quantitative contribution to the total phenolic pool of P3 is minor. The observed COX-2 selectivity of the extract therefore more likely reflects the combined and potentially synergistic action of multiple constituents rather than the activity of a single compound. Kaempferol is best described as a computationally prioritized lead candidate warranting experimental follow-up, not as a confirmed active principle. Despite a marginally higher static docking score (0.1 kcal/mol, within the resolution of the scoring function), apigenin does not display the same dynamic stability and instead shows a partially mismatched binder profile in COX-2, while for COX-1, apigenin behaves comparably to the celecoxib reference and kaempferol exerts a measurable rigidifying effect on the backbone and maintains a tighter pose. As selective COX-2 inhibitors are preferred due to their improved safety profiles compared to COX-1-selective drugs, kaempferol represents the most important active metabolite that acts as COX-2-selective inhibitor among the annotated metabolites and its activity is comparable to that of rofecoxib, the reference drug.

Several limitations of the computational approach must be acknowledged. Molecular docking with AutoDock Vina identifies energetically plausible binding poses and provides a rank-ordered comparison within a congeneric series, but the scoring function does not account for entropic contributions, solvent reorganization, or induced-fit effects beyond the flexibility permitted by the defined rotatable bonds. Trajectory lengths of 200 ns are adequate for comparing closely related scaffolds within the same active site, but may be insufficient to sample rare conformational events or complete ligand dissociation. In addition, the protein models used here are based on crystal structures of the murine COX-2 isoform (PDB: 5KIR) and human COX-1 (PDB: 3KK6); species differences at the binding site are minor and the structures are the highest resolution available for these inhibitor complexes, but the findings should ultimately be validated against the human COX-2 sequence. The computational results presented in this study should be interpreted as mechanistic hypotheses to guide experimental prioritization rather than as quantitative affinity predictions.

A further limitation concerns the validation of the docking protocol. The protocol was tested against positive controls only, in the form of re-docked co-crystallized inhibitors, and was not tested against negative data. Its ability to discriminate binders from nonbinders is consequently unknown, and the ranking of the 26 metabolites should not be read as a prediction of their relative COX inhibitory potency [50,51]. The experimentally measured COX-2 inhibition and selectivity of the P3 extract establish that inhibitory activity is present in the mixture, but the assignment of that activity to any individual constituent remains a computational hypothesis. Testing it requires a bioassay of the isolated compounds, which the docking results are intended to prioritize rather than to replace. The molecular dynamics component is subject to the same constraint. At 200 ns per system, the trajectories are far shorter than the timescale on which ligands of this class dissociate, and no dissociation was observed or expected. Persistence of the bound pose over the sampled window therefore does not indicate that these complexes are stable under experimental conditions [44,52]. The comparisons drawn between systems reflect differences in fluctuation amplitude within the bound state, and are further constrained by force-field limitations that bias simulated structures toward over-stabilization. The dynamic analysis refines a structural hypothesis; it does not test it.

The dynamic performance of kaempferol within the COX-2 pocket can be explained by its dual capacity for competitive binding and redox-mediated enzyme inactivation. Kaempferol undergoes a localized chemical transformation within oxidative inflammatory zones into reactive electrophilic intermediates, such as quinone-methides or 2-benzoyl-2-hydroxy-3(2H)-benzofuranone (BZF) structures. These temporary electrophilic intermediates are highly capable of undergoing spontaneous Michael addition reactions with nucleophilic cysteine or lysine residues situated directly along the enzyme’s internal channel walls [56,57,58,59,60].

## 5. Conclusions

This study identifies Saudi propolis collected from the Fayfa Mountains **(P3)** as an exceptional source of bioactive constituents with potent antioxidant and selective anti-inflammatory activities. Among the four regional propolis samples investigated, **P3** consistently exhibited superior biological performance, demonstrating radical-scavenging activity comparable to that of the reference antioxidant ascorbic acid and potent selective inhibition of COX-2. Comprehensive LC/MS profiling revealed a rich phytochemical composition comprising 26 annotated secondary metabolites, predominantly flavonoids and phenolic compounds, which likely underpin its observed pharmacological effects. Furthermore, molecular docking and molecular dynamics simulations provided mechanistic insights into its anti-inflammatory activity, identifying kaempferol as a key bioactive constituent capable of forming a highly stable and persistent complex with COX-2, surpassing the reference inhibitor rofecoxib in overall system stability. Collectively, the integration of biological, chemical, and computational evidence highlights Fayfa Mountain propolis as a valuable natural reservoir of anti-inflammatory compounds and provides a strong scientific rationale for its traditional use. Given the central role of oxidative stress and chronic inflammation in the pathogenesis of numerous contemporary diseases, including cardiovascular, metabolic, neurodegenerative, and malignant disorders, **P3** propolis represents a promising candidate for further preclinical and clinical development as a natural therapeutic and a potential source of novel anti-inflammatory lead compounds. Furthermore, molecular docking and molecular dynamics simulations provided mechanistic hypotheses regarding the anti-inflammatory activity of the annotated metabolites, identifying kaempferol as a computationally prioritized candidate that forms a dynamically stable complex with COX-2. These computational findings are predictive in nature and require experimental validation through a bioassay of isolated compounds, fractionation studies, and mechanistic binding experiments before any constituent can be identified as a confirmed active principle of P3.

## Figures and Tables

**Table 1:** The IC_50_ values of DPPH and ABTS radical-scavenging activities.

Compound	DPPH * IC_50_ (µg/mL)	ABTS * IC_50_ (µg/mL)
P1	84.16 ± 3.13 ^a^	121.84 ± 4.53 ^a^
P2	56.88 ± 2.12 ^c^	109.22 ± 3.46 ^b^
P3	25.84 ± 0.96 ^d^	32.30 ± 1.2 ^d^
P4	66.67 ± 2.48 ^b^	79.05 ± 2.94 ^c^
**Ascorbic acid**	**27.45 ± 1.42 ^d^**	**21.22 ± 0.79 ^e^**

* Data are represented as mean ± SD values (*n* = 3). Means with different superscripts (a, b, c, d, e) between treatments in the same column are significantly different at *p* < 0.05 (mean comparisons were executed using (ANOVA), followed by Tukey’s post hoc test).

**Table 2:** Anti-inflammatory activity of propolis extracts against both COX-1 and COX-2 enzymes.

Compound	COX1/2 * IC50 (µg/mL)		COX-1/COX-2 Ratio
	COX1	COX2	
P1	7.733 ± 0.26 ^a^	17.2 ± 0.58 ^c^	0.449
P2	36.43 ± 1.23 ^c^	25.78 ± 0.87 ^d^	1.413
P3	23.6 ± 0.79 ^b^	6.194 ± 0.21 ^b^	3.810
P4	42.89 ± 1.44 ^d^	15.23 ± 0.51 ^c^	2.816
**Celecoxib**	**24.56 ± 0.83 ^b^**	**0.681 ± 0.02 ^a^**	**36.06**

* Data are represented as mean ± SD values (*n* = 3). **Means with different superscripts (a, b, c, d) between treatments in the same column are significantly different at *p* < 0.05** (mean comparisons were executed using (ANOVA), followed by Tukey’s post hoc test).

**Table 3:** The metabolites dereplicated from the **P3** propolis extract using LC/MS technique.

#	Compound	Mode	Formula	Structure	Class	*m*/*z*	Rt	% vol.	Ref.
**1**	Chlorogenic acid	−	C_16_H_18_O_9_	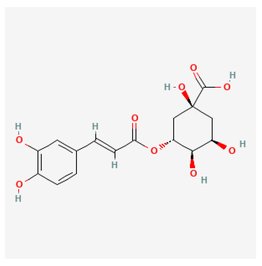	Phenolic acid	353.0973	1.741	8.07%	[33,34,35]
**2**	Caffeic acid	−	C_9_H_8_O_4_	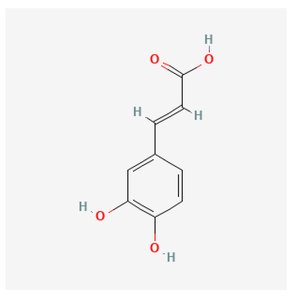	Phenolic acid	179.043	2.533	0.49%	[33,34,35]
**3**	Kaempferol methyl ether	−	C_16_H_12_O_6_	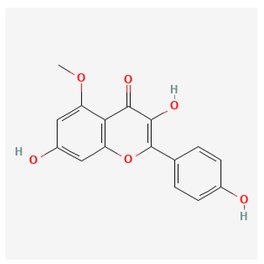	Flavonoid derivatives	299.203	22.84	0.15%	[33,36]
**4**	Galangin-5-methyl ether	-	C_16_H_12_O_5_	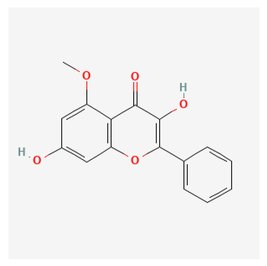	Flavonoid derivatives	283.068	12.42	0.1%	[33,37]
**5**	3,4-Dimethyl-caffeic acid	−	C_11_H_12_O_4_	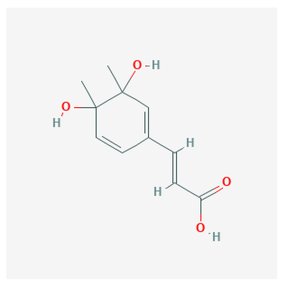	Phenolic acid derivatives	207.110	13.54	0.39%	[33,35]
**6**	Chrysin	−	C_15_H_10_O_4_	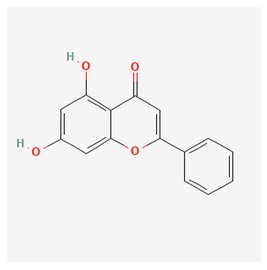	Flavonoid	253.051	14.04	0.24%	[33,35,38]
**7**	Apigenin	−	C_15_H_10_O_5_	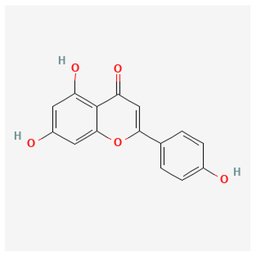	Flavonoid	269.046	14.7	0.41%	[33,35,38]
**8**	Pinobanksin	−	C_15_H_12_O_5_	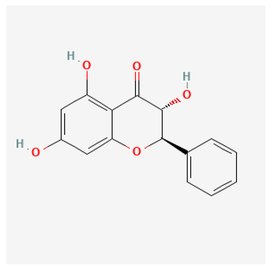	Flavonoid	271.229	18.66	1.36%	[35,39]
**9**	Pinocembrin	−	C_15_H_12_O_4_	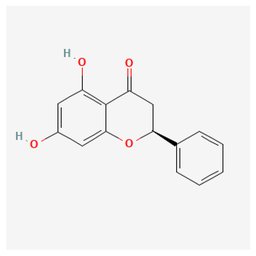	Flavonoid	255.234	28.9	1.11%	[35,39]
**10**	Glycerol monolinolate	−	C_21_H_38_O_5_	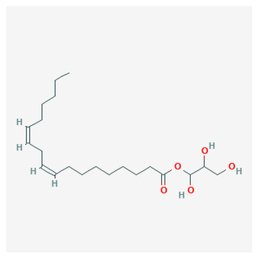	Derivative of linoleic acid	369.245	29.36	1.8%	[40]
**11**	Glyceryl ricinoleate	−	C_21_H_40_O_5_	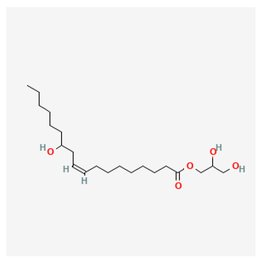	Ester of glycerol and ricinoleic acid	371.261	30.99	2.86%	[41]
**12**	kampherol	+	C_15_H_10_O_6_	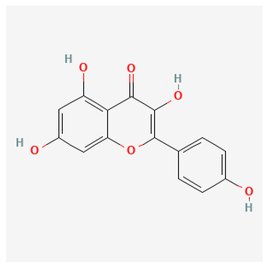	Flavonoid	287.060	9.552	0.02%	[33,35,38,39]
**13**	Ferulic acid	+	C_10_H_10_O_4_	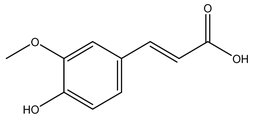	Phenolic acid	195.102	16.84	0.16%	[33,35,38,39,42]
**14**	Dicaffeoylquinic acid	+	C_25_H_24_O_12_	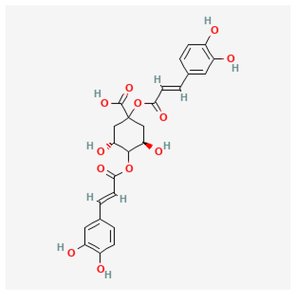	Phenolic acid derivatives	517.288	22.29	0.45%	[33,35,37,39,42]
**15**	Cinnamic acid	+	C_9_H_8_O_2_	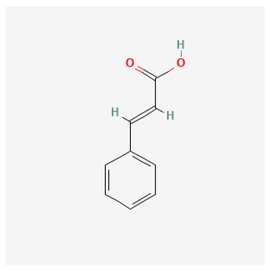	Phenolic acid	149.133	23.6	0.09%	[33,35,37,39,42]
**16**	Epigallocatechin gallate	+	C_22_H_18_O_11_	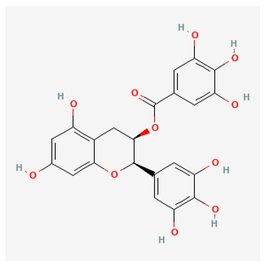	Flavonoid derivatives	459.311	23.6	0.56%	[33,35,37,39,42]
**17**	p-coumaric acid	+	C_9_H_8_O_3_	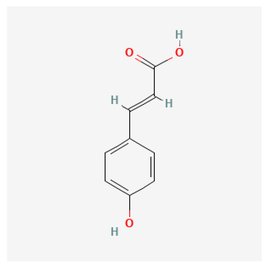	Phenolic acid	165.051	39.49	0.16%	[33,35,37,39,42]
**18**	Stearamide	+	C_18_H_37_NO	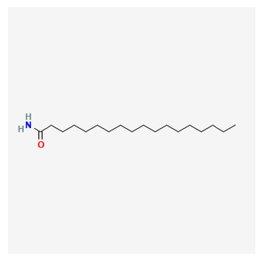	Fatty acid amides	284.296	29.88	2.66%	[43]
**19**	Fumaric aid	+	C_4_H_4_O_4_	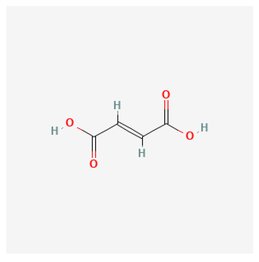	Dicarboxylic acids	217.039	39.67	1.44%	[33,35,37,39,42]
**20**	Protocatechuic aldehyde	+	C_7_H_6_O_3_	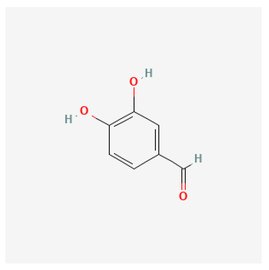	Phenolic acid	138.995	39.65	1.36%	[33,35,37,39,42]
**21**	Epicatechin gallate	+	C_22_H_18_O_10_	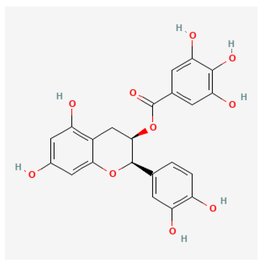	Phenolic	443.389	23.87	0.16%	[33,34,35,36]
**22**	Sinapic acid	+	C_11_H_12_O_5_	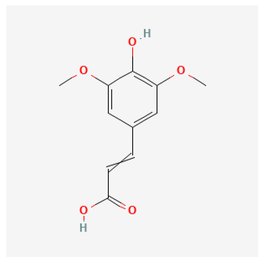	Phenolic acid	225.149	20.67	0.24%	[33,35]
**23**	Miquelianin	+	C_21_H_18_O	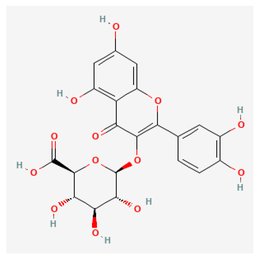	Flavonol glucuronide	479.332	26.83	3.43%	[33,34,35]
**24**	Genistin	+	C_21_H_20_O_10_	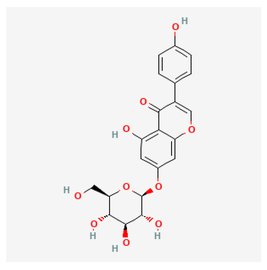	Flavonoid glycoside	433.398	38.87	0. 1%	[34,35]
**25**	Nicotiflorin	+	C_27_H_30_O_15_	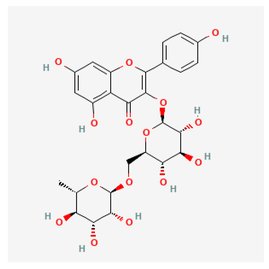	Flavonoid glycoside	595.168	3.29	0.16%	[34,35]
**26**	Naringenin	+	C_15_H_12_O_5_	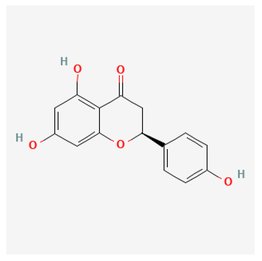	Flavonoid	273.259	23.6	2.0%	[34,35]

**Table 4:** Docking scores and key intermolecular interactions of the **P3** propolis metabolites with COX-1 (PDB ID: 3KK6).

No.	Compound	Chemical Class	Docking Score (kcal/mol)	Hydrogen-Bonding Contacts	Hydrophobic/π Contacts	Other Interactions
**1**	Chlorogenic acid	*Phenolic acid*	**−7.7**	Arg120, Ile517, Ser530; (C–H) Ile523, Leu352	π–σ Phe518	—
**2**	Caffeic acid	*Phenolic acid*	**−7.5**	Met522, Gly526, Ser530, Ile523	π–σ Leu352; π–alkyl Val349, Ala527	—
**3**	Kaempferol methyl ether	*Flavonoid derivative*	**−8.7**	Ile523	π–σ Leu352; alkyl/π–alkyl Ala527, Val349, Phe518, Phe381, Phe205, Tyr385, Leu384, Leu531, Tyr355, Leu359	Amide–π Gly526
**4**	Galangin-5-methyl ether	*Flavonoid derivative*	**−8.9**	Ile523	π–σ Leu352; alkyl/π–alkyl Val349, Ala527, Leu531, Leu359, Phe518, Leu384, Tyr385, Trp387	Amide–π Gly526
**5**	3,4-Dimethyl-caffeic acid	*Phenolic acid derivative*	**−6.8**	His90; (C–H) Ser530	π–alkyl Ala527, Val349, Ile523, Leu352	—
**6**	Chrysin	*Flavonoid*	**−8.8**	Ser530; (C–H) Ala527	π–σ Ile523; π–alkyl Val349, Leu352, Leu531, Tyr355	—
**7**	Apigenin	*Flavonoid*	**−9.2**	(C–H) Ala527, Ser530	π–σ Ile523; π–alkyl Val349, Leu352, Leu531, Tyr355	—
**8**	Pinobanksin	*Flavonoid*	**−8.4**	(C–H) Ile523	π–alkyl Ala527, Val349, Leu352, Leu359, Leu531, Tyr355	Amide–π Gly526
**9**	Pinocembrin	*Flavonoid*	**−8.8**	—	π–σ Ile523, Tyr355; π–alkyl Val349, Ala527, Leu531	—
**10**	Glycerol monolinolate	*Linoleic-acid derivative*	**−7.5**	Arg120	alkyl/π–alkyl Val349, Leu352, Tyr355, Tyr385, Leu531, Phe518, Phe205, Phe209, Phe381, Val344, Val228, Leu534, Gly533, Ala527, Trp387, Leu384	—
**11**	Glyceryl ricinoleate	*Glycerol/ricinoleic-acid ester*	**−7.3**	Arg120, Glu524; (C–H) Pro86/Ile89	alkyl/π–alkyl Val349, Leu352, Leu359, Leu531, Ile523, Ala527, Val116, Tyr355, Phe518, Tyr348, Met522, Trp387, His90, Leu93	—
**12**	Kaempferol	*Flavonoid*	**−9.2**	His90, Ala527	π–σ Ile523; π–alkyl Val349, Leu352, Leu531, Tyr355, Tyr385	—
**13**	Ferulic acid	*Phenolic acid*	**−7.7**	—	π–σ Leu352; π–alkyl Val349, Ala527, Leu531	—
**14**	Dicaffeoylquinic acid	*Phenolic acid derivative*	**−6.3**	Tyr355, Leu359; (C–H) Ser530	alkyl/π–alkyl Val116, Val349, Tyr348, Leu89/Leu93	π–cation Arg120; π–π T-shaped Tyr348
**15**	Cinnamic acid	*Phenolic acid*	**−6.8**	His90, Ile517	π–σ Ile523; π–alkyl Leu352, Ala527	—
**16**	Epigallocatechin gallate	*Flavonoid derivative*	**−8.1**	Tyr385, Arg120, Ile517, His90, Ser353	π–σ Phe518; alkyl/π–alkyl Val116, Val349, Leu531, Leu352, Ala527	—
**17**	p-Coumaric acid	*Phenolic acid*	**−7.1**	Ser530	π–σ Leu352; π–alkyl Val349, Ala527	—
**18**	Stearamide	*Fatty-acid amide*	**−7.0**	—	alkyl/π–alkyl Leu534, Phe205, Trp387, Val349, Tyr385, Tyr348, Leu384, Phe518, Leu352	—
**19**	Fumaric acid	*Dicarboxylic acid*	**−4.9**	(C–H) Ile523	—	—
**20**	Protocatechuic aldehyde	*Phenolic acid*	**−5.9**	Gly526, Ser530	π–σ Leu352; π–alkyl Val349, Ala527	Amide–π Gly526
**21**	Epicatechin gallate	*Phenolic*	**−8.8**	Tyr385, Arg120, Gln192	π–σ Ile523; alkyl/π–alkyl Ala527, Val349, Val116, Leu352, Leu531, Leu359, Phe518	Unfavorable donor–donor (Tyr355)
**22**	Sinapic acid	*Phenolic acid*	**−7.7**	Ser530	π–σ Leu352; alkyl/π–alkyl Val349, Ala527, Met522, Trp387, Phe518	—
**23**	Miquelianin	*Flavonol glucuronide*	**−8.4**	Gln192, His90, Tyr385, Ala527	π–σ Ile523; alkyl/π–alkyl Val349, Leu352, Leu531, Leu359, Tyr355	—
**24**	Genistin	*Flavonoid glycoside*	**−7.3**	Tyr355, Leu93	π–σ Ile523; π–alkyl Val349, Leu352, Val116, Ala527	π–cation Arg120; amide–π Gly526; unfavorable donor–donor
**25**	Nicotiflorin	*Flavonoid glycoside*	**−5.3**	Ile517, Tyr355, Arg120, Leu93	π–σ Ile523, Ser353; π–alkyl Leu352, Phe518, Val116, Val119	—
**26**	Naringenin	*Flavonoid*	**−6.8**	Arg120, Met522	π–σ Leu352; π–alkyl Val349, Ala527, Ile523	π–π T-shaped Tyr355
*	**Celecoxib**	*Reference (re-docked)*	**−8.1**	Ser516, Phe518, Gln192	π–σ Ile523, Ser353; alkyl/π–alkyl Val116, Val349, Ala527, Leu352, Leu359, Leu384, Leu531, Trp387, Tyr385	π–sulfur His90; π–alkyl Ile517

**Table 5:** Docking scores and key intermolecular interactions of the 26 **P3** propolis metabolites with COX-2 (PDB ID: 5KIR).

No.	Compound	Chemical Class	Docking Score (kcal/mol)	Hydrogen-Bonding Contacts	Hydrophobic/π Contacts	Other Interactions
**1**	Chlorogenic acid	*Phenolic acid*	**−7.4**	His90, Phe518, Gln192, Leu352, Val349, Ala527; (C–H) Tyr355, Ser353	π–alkyl Val523, Ala516	—
**2**	Caffeic acid	*Phenolic acid*	**−7.6**	Ser353, Met522; (C–H) His90	π–alkyl Val523, Ala527	—
**3**	Kaempferol methyl ether	*Flavonoid derivative*	**−9.1**	Val523; (C–H) Gly526	alkyl/π–alkyl Val349, Ala527, Leu352, Leu384, Tyr385, Trp387	π–cation Arg120; π–lone pair Ser530
**4**	Galangin-5-methyl ether	*Flavonoid derivative*	**−9.2**	Val523, Ser530	alkyl/π–alkyl Val349, Ala527, Leu352, Leu359, Leu531, Met522, Met113, Tyr385, Phe381, Trp387	π–cation Arg120; π–lone pair Ser530
**5**	3,4-Dimethyl-caffeic acid	*Phenolic acid derivative*	**−6.2**	(C–H) Ser530	alkyl/π–alkyl Val349, Ala527, Val523, Leu352, Phe518	—
**6**	Chrysin	*Flavonoid*	**−9.2**	(C–H) Ala527	π–alkyl Val523, Val349, Tyr355, Leu359	π–cation Arg120
**7**	Apigenin	*Flavonoid*	**−10.3**	(C–H) Ala527, Tyr355	π–alkyl Val523, Val349, Leu531	π–cation Arg120
**8**	Pinobanksin	*Flavonoid*	**−8.9**	Ser530; (C–H) Phe518	π–alkyl Val349, Ala527, Tyr355, Leu352, Leu531	π–cation Arg120; amide–π Gly526; π–donor H-bond Phe518
**9**	Pinocembrin	*Flavonoid*	**−9.1**	(C–H) Ala527, Ser530	π–alkyl Val523, Val349	—
**10**	Glycerol monolinolate	*Linoleic-acid derivative*	**−7.2**	Arg120; (C–H) Ser353	alkyl/π–alkyl Val523, Val349, Met522, Phe518, Tyr348, Tyr385, Tyr115, Phe381, Phe205, Phe209, Leu352, Leu384, Leu534, Val344, Val228, Gly533, Trp387	—
**11**	Glyceryl ricinoleate	*Glycerol/ricinoleic-acid ester*	**−7.2**	Arg120, Arg513, Glu524; (C–H) Pro86	alkyl/π–alkyl Val523, Val349, Tyr355, Ala527, Leu531, Val89, Leu93	—
**12**	Kaempferol	*Flavonoid*	**−10.2**	Ala527; (C–H) Ser353, Ser530	π–σ Val523; π–alkyl Val349, Tyr355, Leu531, Leu359	π–cation Arg120
**13**	Ferulic acid	*Phenolic acid*	**−7.2**	Leu352; (C–H) Ser530	alkyl/π–alkyl Val349, Val523, Ala527, Leu531	Amide–π Gly526
**14**	Dicaffeoylquinic acid	*Phenolic acid derivative*	**−6.2**	Tyr355, Ser119, Ser530; (C–H) Ser353	alkyl/π–alkyl Val349, Val89, Val116, Tyr115	π–cation Arg120; π–π T-shaped Tyr348; π–lone pair Ser530
**15**	Cinnamic acid	*Phenolic acid*	**−6.1**	Phe518, Ile517	π–alkyl Val523, Ala527	—
**16**	Epigallocatechin gallate	*Flavonoid derivative*	**−8.2**	Arg513, Thr94, Ser353, Tyr385, His90, Gln192, Leu352	π–alkyl Val523, Val349, Ala527	—
**17**	p-Coumaric acid	*Phenolic acid*	**−7.3**	Ser530, Leu352; (C–H) Phe518	π–alkyl Val523, Ala527	—
**18**	Stearamide	*Fatty-acid amide*	**−7.1**	(C–H) Ser530	alkyl/π–alkyl Val523, Val349, Val344, Val228, Tyr348, Tyr385, Phe205, Phe209, Phe381, Phe518, Phe529, Leu352, Leu384, Leu534, Trp387, Met522, Ala527	—
**19**	Fumaric acid	*Dicarboxylic acid*	**−4.5**	Leu352; (C–H) Val523	—	—
**20**	Protocatechuic aldehyde	*Phenolic acid*	**−6.1**	(C–H) Gly526	π–alkyl Val523, Val349, Ala527	Amide–π Gly526
**21**	Epicatechin gallate	*Phenolic*	**−8.6**	Tyr385, Tyr355, His90, Gln192	alkyl/π–alkyl Val523, Val349, Ala527, Leu352, Leu531	—
**22**	Sinapic acid	*Phenolic acid*	**−7.7**	Ser530; (C–H) Gly526, His90	alkyl/π–alkyl Val523, Val349, Ala527, Leu352, Leu531, Met522, Phe518, Trp387	—
**23**	Miquelianin	*Flavonol glucuronide*	**−8.6**	Tyr385	π–alkyl Val523, Val349, Leu531, Ala527	π–cation Arg120
**24**	Genistin	*Flavonoid glycoside*	**−7.2**	Arg120, Glu524	π–alkyl Val523, Val349, Ala527, Val89, Leu352	Amide–π Gly526
**25**	Nicotiflorin	*Flavonoid glycoside*	**−5.2**	Arg120, His90, Ile517, Phe518, Gln192, Leu352	π–alkyl Val523, Val89, Tyr115, Ala516	π–lone pair Tyr355/Ser353
**26**	Naringenin	*Flavonoid*	**−6.9**	Arg120; (C–H) Gly526	π–alkyl Val523, Val349, Ala527, Leu352, Leu531	π–π T-shaped Tyr355
*	**Rofecoxib**	*Reference (re-docked)*	**−10.4**	His90, Arg513; (C–H) Ala527	π–alkyl Val523, Val349, Leu352	Amide–π Phe518

## Data Availability

The original contributions presented in this study are included in the article/Supplementary Materials. Further inquiries can be directed to the corresponding authors.

## Supplementary Materials

The following supporting information can be downloaded at: https://www.mdpi.com/article/10.3390/pharmaceutics18091050/s1, Table S1. Key binding interaction distances (Å) for all P3 metabolites docked into COX-1 (PDB: 3KK6). Table S2. Key binding interaction distances (Å) for all P3 metabolites docked into COX-2 (PDB: 5KIR).
