# Supplementary material for: Exploring the Anti-Inflammatory Potential of Saudi Propolis Through Phytochemical Characterization, Molecular Docking, and Dynamic Simulation
Source: Pharmaceutics. 2026 Aug 24;18(9):1050. doi: 10.3390/pharmaceutics18091050 (PMC13610358; doi:10.3390/pharmaceutics18091050)
Supplement: Supplementary file 1 [file pharmaceutics-18-01050-s001.zip › pharmaceutics-4477614-supplementary.pdf]

Supplementary Table S1. Key binding interaction distances (Å) for all P3 metabolites docked into COX-1 (PDB: 3KK6).

No.	Compound	Key interaction distances (Å)
**1**	Chlorogenic acid	H-bond Arg120: 3.12; H-bond Ser530: 3.24; C–H Ile523: 3.58; C–H Leu352: 3.71
**2**	Caffeic acid	H-bond Met522: 3.18; H-bond Ser530: 3.21; π-σ Leu352: 3.82; π-alkyl Val349: 4.09
**3**	Kaempferol methyl ether	C–H Ile523: 3.54; π-σ Leu352: 3.76; π-alkyl Ala527: 4.04; π-alkyl Val349: 4.11; amide-π Gly526: 3.91
**4**	Galangin-5-methyl ether	C–H Ile523: 3.51; π-σ Leu352: 3.79; π-alkyl Val349: 4.08; π-alkyl Ala527: 4.03; amide-π Gly526: 3.88
**5**	3,4-Dimethyl-caffeic acid	H-bond His90: 3.09; C–H Ser530: 3.64; π-alkyl Ala527: 4.07; π-alkyl Val349: 4.13
**6**	Chrysin	H-bond Ser530: 3.19; C–H Ala527: 3.61; π-σ Ile523: 3.74; π-alkyl Val349: 4.06
**7**	Apigenin	C–H Ala527: 3.52; C–H Ser530: 3.61; π-σ Ile523: 3.78; π-alkyl Val349: 4.12; π-alkyl Leu352: 4.05
**8**	Pinobanksin	C–H Ile523: 3.57; π-alkyl Ala527: 4.02; π-alkyl Val349: 4.09; amide-π Gly526: 3.93
**9**	Pinocembrin	π-σ Ile523: 3.77; π-σ Tyr355: 3.83; π-alkyl Val349: 4.10; π-alkyl Ala527: 4.06
**10**	Glycerol monolinolate	H-bond Arg120: 3.08; π-alkyl Val349: 4.14; π-alkyl Leu352: 4.07; π-alkyl Tyr355: 4.21
**11**	Glyceryl ricinoleate	H-bond Arg120: 3.11; H-bond Glu524: 3.17; C–H Pro86: 3.68; π-alkyl Val349: 4.09
**12**	Kaempferol	H-bond His90: 2.94; H-bond Ala527: 3.08; π-σ Ile523: 3.71; π-alkyl Val349: 4.09; π-alkyl Leu352: 4.01
**13**	Ferulic acid	π-σ Leu352: 3.81; π-alkyl Val349: 4.11; π-alkyl Ala527: 4.08; π-alkyl Leu531: 4.19
**14**	Dicaffeoylquinic acid	H-bond Tyr355: 3.14; H-bond Leu359: 3.22; C–H Ser530: 3.67; π-cation Arg120: 3.88
**15**	Cinnamic acid	H-bond His90: 3.16; H-bond Ile517: 3.24; π-σ Ile523: 3.80; π-alkyl Leu352: 4.07
**16**	Epigallocatechin gallate	H-bond Tyr385: 2.98; H-bond Arg120: 3.06; H-bond His90: 3.11; H-bond Ser353: 3.19; π-σ Phe518: 3.77
**17**	p-Coumaric acid	H-bond Ser530: 3.22; π-σ Leu352: 3.83; π-alkyl Val349: 4.10; π-alkyl Ala527: 4.06
**18**	Stearamide	π-alkyl Val349: 4.13; π-alkyl Leu352: 4.08; π-alkyl Tyr385: 4.24; π-alkyl Trp387: 4.31
**19**	Fumaric acid	C–H Ile523: 3.71
**20**	Protocatechuic aldehyde	H-bond Gly526: 3.27; H-bond Ser530: 3.31; π-σ Leu352: 3.84; amide-π Gly526: 3.94
**21**	Epicatechin gallate	H-bond Tyr385: 2.96; H-bond Arg120: 3.04; H-bond Gln192: 3.13; π-σ Ile523: 3.73
**22**	Sinapic acid	H-bond Ser530: 3.20; π-σ Leu352: 3.80; π-alkyl Val349: 4.08; π-alkyl Phe518: 4.17
**23**	Miquelianin	H-bond Gln192: 3.09; H-bond His90: 3.14; H-bond Tyr385: 3.01; H-bond Ala527: 3.11; π-σ Ile523: 3.72
**24**	Genistin	H-bond Tyr355: 3.17; H-bond Leu93: 3.31; π-σ Ile523: 3.75; π-cation Arg120: 3.86
**25**	Nicotiflorin	H-bond Ile517: 3.22; H-bond Tyr355: 3.18; H-bond Arg120: 3.07; π-σ Ile523: 3.76
**26**	Naringenin	H-bond Arg120: 3.13; H-bond Met522: 3.26; π-σ Leu352: 3.82; π-π T-shaped Tyr355: 3.94
*****	Celecoxib (reference)	H-bond Ser516: 2.88; H-bond Phe518: 3.21; H-bond Gln192: 3.14; π-sulfur His90: 3.85; π-σ Ile523: 3.69

Supplementary Table S2. Key binding interaction distances (Å) for all P3 metabolites docked into COX-2 (PDB: 5KIR).

No.	Compound	Key interaction distances (Å)
**1**	Chlorogenic acid	H-bond His90: 3.07; H-bond Phe518: 3.19; H-bond Gln192: 3.11; π-alkyl Val523: 4.08
**2**	Caffeic acid	H-bond Ser353: 3.21; H-bond Met522: 3.17; C–H His90: 3.63; π-alkyl Val523: 4.05
**3**	Kaempferol methyl ether	C–H Val523: 3.56; C–H Gly526: 3.62; π-alkyl Val349: 4.07; π-alkyl Ala527: 4.03; π-cation Arg120: 3.81
**4**	Galangin-5-methyl ether	H-bond Val523: 3.24; H-bond Ser530: 3.19; π-alkyl Val349: 4.05; π-alkyl Ala527: 4.01; π-cation Arg120: 3.77
**5**	3,4-Dimethyl-caffeic acid	C–H Ser530: 3.64; π-alkyl Val349: 4.09; π-alkyl Val523: 4.06; π-alkyl Leu352: 4.13
**6**	Chrysin	C–H Ala527: 3.58; π-alkyl Val523: 4.04; π-alkyl Val349: 4.11; π-cation Arg120: 3.83
**7**	Apigenin	C–H Ala527: 3.48; C–H Tyr355: 3.55; π-alkyl Val523: 4.06; π-alkyl Val349: 4.14; π-cation Arg120: 3.84
**8**	Pinobanksin	H-bond Ser530: 3.16; C–H Phe518: 3.59; π-alkyl Val349: 4.08; π-alkyl Ala527: 4.04; π-cation Arg120: 3.86
**9**	Pinocembrin	C–H Ala527: 3.53; C–H Ser530: 3.61; π-alkyl Val523: 4.07; π-alkyl Val349: 4.12
**10**	Glycerol monolinolate	H-bond Arg120: 3.09; C–H Ser353: 3.66; π-alkyl Val523: 4.04; π-alkyl Val349: 4.13
**11**	Glyceryl ricinoleate	H-bond Arg120: 3.06; H-bond Arg513: 3.11; H-bond Glu524: 3.18; C–H Pro86: 3.71; π-alkyl Val523: 4.02
**12**	Kaempferol	H-bond Ala527: 3.02; C–H Ser353: 3.62; C–H Ser530: 3.58; π-σ Val523: 3.74; π-alkyl Val349: 4.08; π-cation Arg120: 3.79
**13**	Ferulic acid	H-bond Leu352: 3.23; C–H Ser530: 3.67; π-alkyl Val349: 4.10; π-alkyl Val523: 4.07; amide-π Gly526: 3.91
**14**	Dicaffeoylquinic acid	H-bond Tyr355: 3.16; H-bond Ser119: 3.22; H-bond Ser530: 3.19; π-cation Arg120: 3.87; π-π T-shaped Tyr348: 3.96
**15**	Cinnamic acid	H-bond Phe518: 3.24; H-bond Ile517: 3.28; π-alkyl Val523: 4.09; π-alkyl Ala527: 4.06
**16**	Epigallocatechin gallate	H-bond Arg513: 3.04; H-bond Thr94: 3.11; H-bond Ser353: 3.17; H-bond Tyr385: 2.97; H-bond His90: 3.08; π-alkyl Val523: 4.03
**17**	p-Coumaric acid	H-bond Ser530: 3.21; H-bond Leu352: 3.27; C–H Phe518: 3.61; π-alkyl Val523: 4.05
**18**	Stearamide	C–H Ser530: 3.66; π-alkyl Val523: 4.04; π-alkyl Val349: 4.11; π-alkyl Phe518: 4.18
**19**	Fumaric acid	H-bond Leu352: 3.34; C–H Val523: 3.74
**20**	Protocatechuic aldehyde	C–H Gly526: 3.68; π-alkyl Val523: 4.07; π-alkyl Val349: 4.12; amide-π Gly526: 3.92
**21**	Epicatechin gallate	H-bond Tyr385: 2.99; H-bond Tyr355: 3.08; H-bond His90: 3.12; H-bond Gln192: 3.16; π-alkyl Val523: 4.04
**22**	Sinapic acid	H-bond Ser530: 3.18; C–H Gly526: 3.63; C–H His90: 3.69; π-alkyl Val523: 4.06; π-alkyl Ala527: 4.03
**23**	Miquelianin	H-bond Tyr385: 3.03; π-alkyl Val523: 4.05; π-alkyl Val349: 4.12; π-alkyl Leu531: 4.19; π-cation Arg120: 3.88
**24**	Genistin	H-bond Arg120: 3.08; H-bond Glu524: 3.14; π-alkyl Val523: 4.06; π-alkyl Val349: 4.09; amide-π Gly526: 3.89
**25**	Nicotiflorin	H-bond Arg120: 3.11; H-bond His90: 3.09; H-bond Ile517: 3.24; H-bond Phe518: 3.27; π-alkyl Val523: 4.08
**26**	Naringenin	H-bond Arg120: 3.13; C–H Gly526: 3.66; π-alkyl Val523: 4.04; π-alkyl Val349: 4.09; π-π T-shaped Tyr355: 3.92
*****	Rofecoxib (reference)	H-bond His90: 2.91; H-bond Arg513: 3.05; C–H Ala527: 3.44; amide-π Phe518: 3.81; π-alkyl Val523: 4.03
